# *De novo* Prediction of Moonlighting Proteins Using Multimodal Deep Ensemble Learning

**DOI:** 10.3389/fgene.2021.630379

**Published:** 2021-03-22

**Authors:** Ying Li, Jianing Zhao, Zhaoqian Liu, Cankun Wang, Lizheng Wei, Siyu Han, Wei Du

**Affiliations:** ^1^Key Laboratory of Symbol Computation and Knowledge Engineering of Ministry of Education, College of Computer Science and Technology, Jilin University, Changchun, China; ^2^Department of Biomedical Informatics, College of Medicine, Ohiostate University, Columbus, OH, United States; ^3^Department of Computer Science, Faculty of Engineering University of Bristol, Bristol, United Kingdom

**Keywords:** protein moonlighting, ensemble learning, deep learning, multimodal, prediction model

## Abstract

Moonlighting proteins (MPs) are a special type of protein with multiple independent functions. MPs play vital roles in cellular regulation, diseases, and biological pathways. At present, very few MPs have been discovered by biological experiments. Due to the lack of data sample, computation-based methods to identify MPs are limited. Currently, there is no *de-novo* prediction method for MPs. Therefore, systematic research and identification of MPs are urgently required. In this paper, we propose a multimodal deep ensemble learning architecture, named MEL-MP, which is the first *de novo* computation model for predicting MPs. First, we extract four sequence-based features: primary protein sequence information, evolutionary information, physical and chemical properties, and secondary protein structure information. Second, we select specific classifiers for each kind of feature. Finally, we apply the stacked ensemble to integrate the output of each classifier. Through comprehensive model selection and cross-validation experiments, it is shown that specific classifiers for specific feature types can achieve superior performance. For validating the effectiveness of the fusion-based stacked ensemble, different feature fusion strategies including direct combination and a multimodal deep auto-encoder are used for comparative purposes. MEL-MP is shown to exhibit superior prediction performance (F-score = 0.891), surpassing the existing machine learning model, MPFit (F-score = 0.784). In addition, MEL-MP is leveraged to predict the potential MPs among all human proteins. Furthermore, the distribution of predicted MPs on different chromosomes, the evolution of MPs, the association of MPs with diseases, and the functional enrichment of MPs are also explored. Finally, for maximum convenience, a user-friendly web server is available at: http://ml.csbg-jlu.site/mel-mp/.

## 1. Introduction

The study of protein functions is a central issue in the post-genomic era. The investigation of protein functions helps elucidate the varying mechanisms of organisms under physiological or pathological conditions, such as heart disease, autoimmune disease, and cancers. Yet, little is known about the functions of proteins due to their high diversity. In recent years, a new type of protein with two or more distinct functions or subcellular locations, called moonlighting proteins (MPs), has been found (Weaver, 1998; Jeffery, 2003, 2004; Jeffery and Wang, 2016). Research has led to the detection of MPs in various species, including reptiles, plants, and mammals. MPs contain enzymes that act as receptors, DNA stabilizers, components of the backbone, transcription factors, secreted cytokines, and proteasome semihydrolases (Jeffery, 2018). Further, MPs appear to be very useful from the perspective of biological research. Proteins with diversity functions have research value from the perspective of organism evolution. During genome reduction, organisms may increase the functional range of a limited set of genes since multifunctional proteins will increase robustness (Ferla et al., 2017; Nishiyama et al., 2019). Recent studies have also proved that multifunctional proteins play an important role in virulence and diseases. Research on MPs can help improve collective understandings of the relationship between health systems and diseases (Henderson and Martin, 2011; Jeffery, 2015; Franco-Serrano et al., 2018). Given the vital role of MPs in biological fields, the systematic study of MPs is an important task for understanding protein functions.

Identifying MPs mainly relies on experimental methods, which are time consuming and expensive. Currently, computation-based method for predicting MPs are limited, with relevant examples being MoonGO (Chapple et al., 2015), MPFit (Khan and Daisuke, 2016), and DextMP (Khan et al., 2017). MoonGO applies statistical methods and an overlapping clustering algorithm based on annotated gene ontology (GO) information. MPFit uses GO annotation (Gene Ontology Consortium et al., 2013) or a combination of information from six omic-based protein associations (protein–protein interaction, gene expression, phylogenetic profiles, genetic interactions, network-based graph properties, and disordered protein regions) to predict MPs. DextMP relies on a natural language processing method to predict MPs based on the published literature, i.e., title, abstract, and function description (Kim et al., 2014). However, not all proteins have the necessary annotation and text information, which leads to a considerable amount of proteins lacking feature representations in the above two existing methods. Therefore, *de novo* computation methods to comprehensively identify MPs are urgently required.

Here, we propose a *de novo* multimodal deep ensemble learning method based on sequences to identify MPs, named MEL-MP. To this end, four types of features are extracted: primary protein sequence information, evolutionary information, physical and chemical properties, and secondary protein structure information. For each kind of feature, we select the optimal classifier based on 10-fold cross validation. Then, each kind of feature is classified by a base classifier, with the best performance as a sub-model. Finally, a stacked ensemble strategy is applied to integrate the outputs of each sub-model. Compared with other feature fusion methods (i.e., direct combination and multimodal deep auto-encoder), MEL-MP exhibits superior performance. After that, MEL-MP is applied to the whole human genome to predict potential MPs. We further explore predicted MPs from four different perspectives: the distribution on human chromosomes, the association with diseases, evolutionary history and the functional analysis. The results reveal that the predicted MPs are significantly related to diseases, and the ratio of MPs in the Y chromosome is higher compared with other chromosomes. Compared with non-MPs, MPs may have earlier origination and show multifunctional nature. In order to facilitate the use of MEL-MP, we have developed a web server with friendly interface. MEL-MP not only contributes a novel *de novo* prediction tool for predicting MPs with satisfactory accuracy to facilitate future research but also provides an enhanced scheme for multimodal feature fusion.

## 2. Materials and Methods

### 2.1. Data Sets

Data sets, including positive and negative samples, are determined as per MPFit. Note that 268 positive samples are selected from the moonprot database released in 2015 (Mathew et al., 2015). These data are verified by biological experiments. However, negative samples are not directly discovered by biological experiments and thus manual construction is required in terms of annotation by GO information. In this task, a protein is determined as non-MP according to the clustering results of GO terms (Khan and Daisuke, 2016). The similarity score of GO terms is calculated to measure the relationship between GO terms using Rel's method (Schlicker et al., 2006). To construct the negative samples, the first step is to calculate the frequency of a single GO term *c* as follows:

(1) $$\begin{array}{r} freq\left(c\right)=anno\left(c\right)+\underset{h\in children\left(c\right)}{\sum }freq\left(h\right) \end{array}$$

where the *anno*(*c*) is the number of gene product annotated with the GO term *c* in the database. *children*(*c*) is the child GO set of the GO term *c*. The freq(c) refers to the frequency of the GO term *c*. *freq*(*c*) is expressed recursively, which means when term *c* has the child set, the frequency of the GO term *c* is the sum of the annotation of GO term *c* itself and all frequency of the childs of GO term *c*. When *c* does not has childs GO terms, the *freq*(*c*) is equal to *anno*(*c*). Then the probability (*p*) of a GO term *c* is defined as follows:

(2) $$\begin{array}{r} p\left(c\right)=freq\left(c\right)/freq\left(root\right) \end{array}$$

where root refers to the ancestor of GO term *c*. Finally, the GO semantic similarity score between GO term *c*_1_ and *c*_2_ is defined as:

(3) $$\begin{array}{r} sim_{Rel\left(c_{1},c_{2}\right)}=\underset{c\in S\left(c_{1},c_{2}\right)}{\max}\left(\frac{2\times \log p\left(c\right)}{\log p\left(c_{1}\right)+\log p\left(c_{2}\right)}\times \left(1-p\left(c\right)\right)\right) \end{array}$$

where *S*(*c*_1_, *c*_2_) is the set of common ancestors of terms *c*_1_ and *c*_2_. Thence, a protein can be determined as a negative sample is based on three principles (Khan and Daisuke, 2016): (1) the protein has at least eight GO terms; (2) the biological process (BP) terms are in one cluster with a similarity score between 0.1 and 0.5; (3) when molecular function (MF) terms are clustered, no more than one cluster reaches a similarity score between 0.1 and 0.5. Utilizing these three principles to screen proteins of four species (human, yeast, *Escherichia coli*, and mouse). After removing the negative samples with the same sequence as the positive samples, 162 proteins are defined as negative samples.

#### 2.1.1. Primary Sequence Feature

We calculate the *k* − *mer* features of protein sequences, which are defined as the numbers of occurrences of all *k* amino acids arrangements in a protein sequence. A protein sequence has 20 different types of amino acid, so the length of the *k* − *mer* vector is equal to 20^*k*^. That is, the length of *k* − *mer* vector is equal to 20^*k*^. Here, we choose *k* = 3, 2, 1, respectively, and then, respectively, concatenate the 3 − *mer*, 2 − *mer*, and 1 − *mer* features of a sequence together as the *rawkmers* feature of a sequence:

(4) $$\begin{array}{r} rawkmers=\left(3-mer,2-mer,1-mer\right) \end{array}$$

Furthermore, to reduce the dimensions and extract informative feature from *rawkmers*, the autocorrelation coefficient function (Acf) is given as follows:

(5) $$r_{j}=\frac{\sum_{i=1}^{n-j}\left(X_{i}-X_{mean}\right)\left(X_{i+j}+X_{mean}\right)}{\sum_{i=1}^{n}{\left(X_{i}-X_{mean}\right)}^{2}},\;j=1,...m$$

(6) $$\begin{array}{r} Seq=\left(r_{1},r_{2},...r_{j},...r_{m}\right) \end{array}$$

where *X* is the *rawkmers*, *n* is the length of *rawkmers*, *X*_*mean*_ represents the mean of *X*, and *j* is an integer ranging from [0, *m*]. Here we select *m* = 400. After pre-processing *rawkmers* through the Acf algorithm, *Seq* is used as the primary sequence feature vector of a sequence.

#### 2.1.2. Evolutionary Information

We extract the position-specific score matrix (*rawpssm*) as evolutionary features through PSI-BLAST (Altschul et al., 1997). A non-redundant protein sequence library, swiss-prot, is used as the sequence alignment database. We run PSI-BLAST with the commonly used hyperparameters (*e*-value is 0.001, and the number of iterations is 3) (Taherzadeh et al., 2017; Le et al., 2019). The generated *rawpssm* of a protein sequence is a *L* × 20 matrix as follows:

(7) $$\begin{array}{r} \left[\begin{array}{cccc} x_{\left(1,1\right)} & x_{\left(1,2\right)} & \cdots & x_{\left(1,20\right)}\\ x_{\left(2,1\right)} & x_{\left(2,2\right)} & \cdots & x_{\left(2,20\right)}\\ \vdots & \vdots & \ddots & \vdots \\ x_{\left(L,1\right)} & x_{\left(L,2\right)} & \cdots & x_{\left(L,20\right)} \end{array}\right] \end{array}$$

where *L* is the length of a given protein sequence and *x*_(*i, j*)_ represents the position specific score. Due to different protein sequences having different lengths, we then extract features with a fixed length from *rawpssm* as the evolutionary feature through **Algorithm 1**. For a given sample, the protein sequence (*sequence*) and rawpssm are inputs, and the output *PSSM* by **Algorithm 1** is used as input to the classification model. *PSSM* is a feature vector with 400 dimensions.

**Algorithm 1:** 

Require: rawpssm, sequence
Ensure: PSSM
1: tag ← [A,C,D,E,F,G,H,I,K,L,M,N,P,Q,R,S,T,V,W,Y]
2: Initialize index, PSSM
3: n ← length(tag)
4: l ← length(sequence)
5: for i=0 → n-1 **do**
6: Initialize num
7: for key=0 → n-1 **do**
8: index[tag[key]] = 0
9: end **for**
10: for j=0 → l-1 **do**
11: index[sequence[j]] + = rawpssm[j, i]
12: end **for**
13: for k=0 → n-1 **do**
14: num[k] ← index[tag[k]]
15: end **for**
16: PSSM[i] ← num
17: end **for**
18: PSSM ← PSSM / l

#### 2.1.3. Physical and Chemical Properties Feature

We obtain physical and chemical information for proteins through the AAindex database (Kawashima S, 2000), which covers 566 physicochemical properties. Thirteen of these physicochemical properties contain missing values. Therefore, we select the other 553 types of amino acid properties to transform the protein sequences into digital sequences:

(8) $$\begin{array}{r} Sub=\left(s_{1},s_{2},......s_{553}\right) \end{array}$$

where *s*_*i*_, (*i* = 1, 2...553) is of a dimension *L* (*L* is sequence length), and it represents the *i*_*th*_ physicochemical property of a protein sequence. Each protein can be expressed as a matrix with a size of (553 × *L*). Given different lengths of protein sequences, we extract the mean and variance of each sequence and concatenate them together as the physical and chemical properties feature vector *AA*:

(9) $$\begin{array}{r} AA=\left(mean_{1},var_{1},mean_{2},var_{2},......mean_{553},var_{553}\right) \end{array}$$

where *mean*_*i*_, *var*_*i*_ refer to the mean and variance of *s*_*i*_, respectively.

#### 2.1.4. Secondary Structure Feature

We compute the secondary structure of a protein by SSpro8 (Cheng et al., 2005), which can generate the 8-class secondary structure sequences, including H (alpha helix), B (residue in isolated beta-bridge), E (extended strand, participates in beta ladder), G (3-helix—3/10 helix), I (5-helix—pi helix), T (hydrogen bonded turn), S (bend), and C (loop or irregular, coil). We then compute *k* − *mers*, (*k* = 3, 2, 1) of the secondary structure sequence and obtain the feature vector of the secondary structure as follows:

(10) $$\begin{array}{r} SS=\left(3-mer,2-mer,1-mer\right) \end{array}$$

### 2.2. Selection of Each Sub-Model

In what follows, we select the appropriate machine learning model to select sub-models for each kind of feature.

#### 2.2.1. Bidirectional Long Short-Term Memory Neural Networks for Primary Protein Sequence Information

Bidirectional long short-term memory neural networks (BiLSTMs) (Hochreiter and Schmidhuber, 1997) is selected to train the feature vector of primary sequence information *Seq*. BiLSTM contains two opposite LSTMs. In the BiLSTM layer, the forward LSTMs generate hidden vectors *h*_*f*_ according to the sequence from left to right for every segment, and the backward LSTM generates hidden vectors *h*_*b*_ according to the sequence from right to left. The *h*_(*f, t*)_ and *h*_(*b, t*)_ represent the output at each time step *t*(*t* = 1, 2, ...*n*) for forward LSTM and backward LSTM, respectively. Then, the joint representation [*h*_(*f, t*)_, *h*_(*b, t*)_] refers to the hidden state of BiLSTM. Finally, the joint vector *h* = [*h*_1_, *h*_2_, ...*h*_*t*_, ...*h*_*n*_] is the output of BiLSTM.

#### 2.2.2. Random Forest for Evolutionary Information

We use random forest (RF) (Breiman, 2001) as our base classifier for PSSM features. RF contains multiple decision trees, and its output is determined by the outputs of individual trees. The grid search method is used to determine the hyperparameters of RF.

#### 2.2.3. Multilayer Perceptron for Physical and Chemical Properties

The multilayer perceptron (MLP) is applied to train physical and chemical properties feature vector *AA*. In the MLP, layers are fully connected, and parameters can be optimized with gradient descent. The relationship between two connected layers is as follows:

(11) $$\begin{array}{r} h_{i+1}=\sigma \left(\sum w_{i}\times h_{i}+b_{i}\right) \end{array}$$

where *h*_*i*_ represents the previous layer, *h*_(_*i* + 1) refers to the next layer, *w*_*i*_ and *b*_*i*_ represent the weight and bias between *h*_*i*_ and *h*_(_*i* + 1), respectively, and σ is a nonlinear activation.

#### 2.2.4. Auto-Encoder and RF for Secondary Protein Structure Information

The secondary structure feature is pre-processed by a deep auto-encoder (Vincent et al., 2010; Vladimir et al., 2018) for high-level feature representation. The auto-encoder is an unsupervised neural network. The middle layer is the encoded data, which extracts the high-dimensional representation of raw data. The architecture of the deep auto-encoder is shown as follows:

(12) $$\begin{array}{r} m=\sigma \left(\sum w_{m}\times x+b_{m}\right) \end{array}$$

(13) $$\begin{array}{r} z=\sigma \left(\sum w_{z}\times m+b_{z}\right) \end{array}$$

where *m*, *z* represent the middle layer and output layer of the auto-encoder, respectively, *w*_*m*_, *w*_*z*_ represent the weight of *m* and *z*, respectively, *b*_*m*_ and *b*_*z*_ represent the bias of *m* and *z*, respectively. The low-dimensional embedding *m* is the input of RF.

### 2.3. Stacked Ensemble

**Figure 1:**
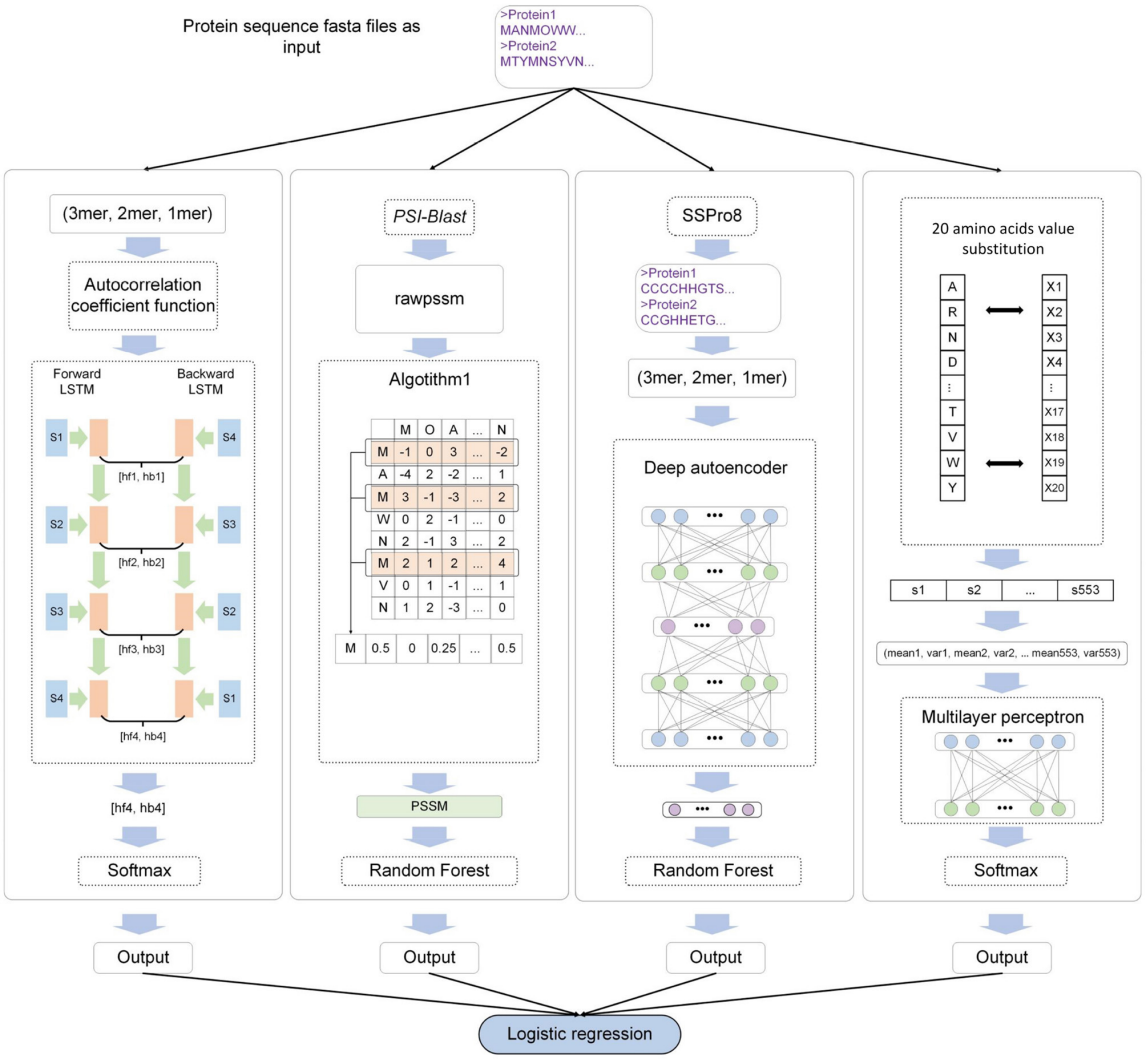
The MEL-MP workflow. The four sub-models correspond to four types of extracted features. *Seq* − *BiLSTM* denotes that the bidirectional long short-term memory neural network (BiLSTM) model is selected for primary sequence features. *PSSM* − *RF* means that the random forest (RF) classifier is used for *PSSM* evolution information. *SS* − *ARF* denotes that the classifier based on an auto-encoder and RF is applied for secondary structure features. *AA* − *MLP* means that the multilayer perceptron (MLP) model is applied for physical and chemical features. Finally, logistic regression (LR) integrates the outputs of all models.

By comparison with other feature fusion methods including direct combination and multimodal deep auto-encoder (Vincent et al., 2010; Vladimir et al., 2018), a stacked ensemble is used. The stacked ensemble strategy can automatically integrate different results of different models. The structure of the stacked ensemble approach contains multiple layers, and the output results of the previous layer will be used as the training data of the next layer. In this way, the next layer will find a suitable combination of individual results from the previous layer. Herein, the output of the first layer is the output prediction label of our four models (*Seq* − *BiLSTM*, *PSSM* − *RF*, *AA* − *MLP*, and *SS* − *ARF*). The second layer is logistic regression (LR), which is applied to integrate the output of the first layer.

(14) $$\phi (z)=\frac{1}{1+exp(\sum_{i=0}^{n}w_{i}\times x_{i}})$$

where *x* represents the output of each sub-model, *w* is the weight of LR, and *n* is the number of input samples. The MEL-MP framework is shown in Figure 1.

### 2.4. 10-Fold Cross-Validation Evaluation

We apply 10-fold cross-validation on the benchmark data set. Note that 90% of samples are used for training and the remaining 10% are used for testing. After the four sub-models are trained, the output of each sub-model is integrated by stacked ensemble in terms of LR. Finally, the test set is applied to quantify the performance of MEL-MP.

The performance of our method is evaluated by Precision, Recall, and F-score. To deal with the problem of the imbalance between positive and negative samples, we use the average weight of the class for every criterion. F-scores combine precision and recall. Therefore, we mainly focus on that particular metric. In addition, the (Bradley, 1997) curve and the coordinate axis (AUC) are provided. For 10-fold cross-validation, all evaluation criteria values are generated by calculating the mean value of each fold. We ensure that there is no repeat of the training and test sets for each fold.

## 3. Results

### 3.1. Sub-Model Selection for Each Feature Type

Various traditional machine learning models and deep learning models are applied to select the suitable sub-model classifier for each kind of feature. Through multiple experiments, sub-models are selected based on 10-fold cross-validation. The results are shown in Table 1. Three single sub-models, *AA* − *MLP*, *SS* − *ARF*, and *Seq* − *BiLSTM*, applied deep learning strategies, and we conducted experiments to select suitable hyperparameters for the neural network models (Supplementary Tables 1–3).

**Table 1:** The performance of all features tested by diverse of methods and ensemble leaning.

**Feature**	**Method**	**Precision**	**Recall**	**F-score**
Sequence	k-mer + LR	0.792	0.743	0.746
	k-mer + RF	0.779	0.773	0.758
	k-mer + SVM	0.767	0.748	0.740
	Acf(k-mer) + LR	0.766	0.755	0.755
	Acf(k-mer) + RF	0.819	0.813	0.812
	Acf(k-mer) + SVM	0.809	0.806	0.803
	**Acf(k-mer)** **+** **BiLSTM**	**0.851**	**0.844**	**0.851**
	Acf(k-mer) + MLP	0.827	0.835	0.827
	Acf(k-mer) + 1DCNN	0.822	0.818	0.814
PSSM	LR	0.829	0.818	0.819
	**RF**	**0.889**	**0.881**	**0.881**
	SVM	0.866	0.862	0.862
	BiLSTM	0.830	0.827	0.824
	MLP	0.866	0.862	0.862
	2DCNN	0.865	0.848	0.850
Physical	LR	0.796	0.792	0.789
chemical	RF	0.843	0.839	0.837
property	SVM	0.843	0.841	0.840
	1DCNN	0.833	0.827	0.824
	BiLSTM	0.837	0.825	0.822
	**MLP**	**0.861**	**0.853**	**0.853**
Secondary	kmer + LR	0.769	0.752	0.755
Structure	kmer + RF	0.797	0.794	0.792
	kmer + SVM	0.806	0.801	0.798
	Autoencoder(kmer) + LR	0.749	0.738	0.738
	**Autoencoder(kmer)+** **RF**	**0.839**	**0.837**	**0.833**
	Autoencoder(kmer) + SVM	0.797	0.792	0.787

*The bold part represents the best classification model for each feature*.

**(1)** Primary protein sequence information

When applying Acf on rawkmers and generating the *Seq*, the accuracy of rawkmers improves. Then LR, RF, support vector machine (SVM) (Khan et al., 2012), 1-dimension convolutional neural network (1DCNN), MLP, and BiLSTM were compared. The BiLSTM classifier has the highest precision, recall, and F-score among the classifiers.

**(2)** Evolution information

Evolutionary information is represented as 400-dimension vectors. The performance of three traditional machine learning classifiers (LR, RF, and SVM) and three deep learning classifiers (BiLSTM, 2-dimension CNN (2DCNN), and MLP) are compared. Results show that the RF model exhibits superior performance. The precision, recall, and F-score associated with this model were 0.889, 0.881, and 0.881, respectively. Thus, RF is used as the sub-model for evolutionary information.

**(3)** Physical and chemical properties

For extracted physical and chemical properties, three traditional machine learning classifiers (LR, RF, and SVM) and three deep learning classifiers (1DCNN, BiLSTM, and MLP) are compared. Results show that MLP is the best classifier among them. The precision, recall, and F-score of MLP are 0.861, 0.853, and 0.853, respectively.

**(4)** The classifier based on the integration of a deep auto-encoder and RF exhibits better performance (precision = 0.839, recall = 0.837, and F-score = 0.833) compared to LR and SVM. In addition, from the results, the auto-encoder used for extracting high-level secondary structure features is effective at learning the informative features and thus improving performance.

### 3.2. Stacked Ensemble Improves the Performance

**Figure 2:**
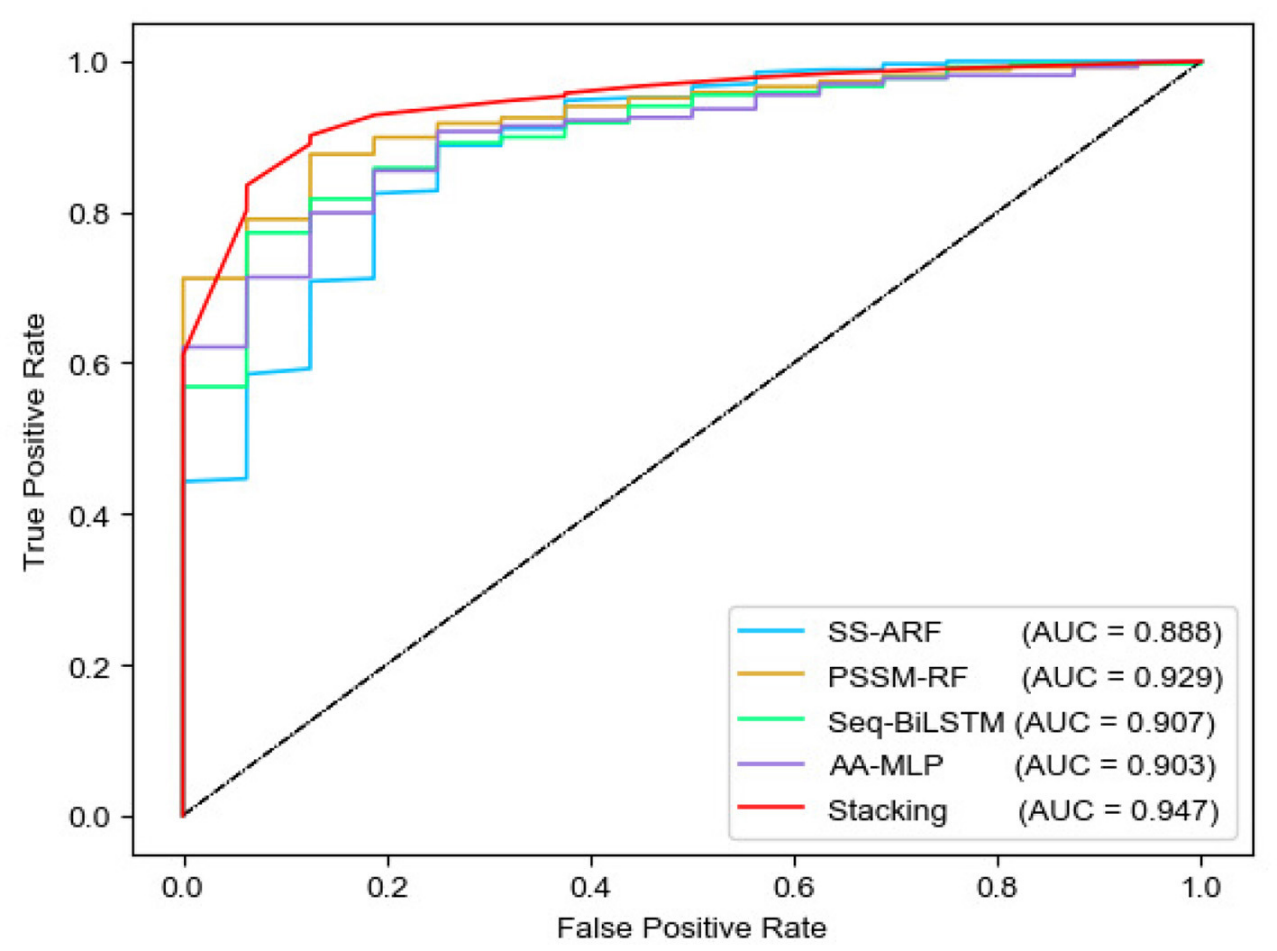
Roc curves of four single model and stacking method.

We analyzed the performance of four sub-models and MEL-MP by 10-fold cross-validation (Tables 1 and 2). The PSSM-RF sub-model achieved the best performance. While using the stacked ensemble strategy, MEL-MP achieved an F-score of 0.892, which is higher than any sub-model. Figure 2 shows ROC curves and AUC values for each sub-model and MEL-MP; the latter is superior.

**Table 2:** Comparison with other feature fusion method and MEL-MP.

**Method**	**Precision**	**Recall**	**F-score**
**Direct combination**			
LR	0.836	0.822	0.823
RF	0.869	0.865	0.865
SVM	0.833	0.830	0.827
Auto-encoder + LR	0.664	0.650	0.651
Auto-encoder + RF	0.787	0.783	0.777
Auto-encoder + SVM	0.767	0.767	0.757
**Multimodal deep auto-encoder**			
Multimodal auto-encoder + LR	0.841	0.832	0.831
Multimodal auto-encoder + RF	0.883	0.881	0.879
Multimodal auto-encoder + SVM	0.860	0.857	0.854
**Stacked ensemble**			
**MEL-MP**	**0.895**	**0.893**	**0.892**

*Each feature fusion method is shown in bold, and the experimental results of MEL-MP are also shown in bold*.

### 3.3. Comparison With Other Feature Fusion Methods

We compared stacked ensemble with other feature fusion strategies, including direct combination and multimodal deep auto-encoder. The stacked ensemble exhibited superior performance based on sequence-based features (Table 2). The architecture and results of other strategies are as follows.

#### 3.3.1. Direct Combination Method

The direct combination method joins the four feature vector types into a new vector for classification. We use the concatenated *Seq*, *PSSM*, *SS*, and *AA* vector to select models in two ways:

**(1)** Using the joint vectors directly as input to the three traditional classifiers, LR, RF, and SVM. Results showed that RF performed best (F-score = 0.865). Use the joint vectors directly as input to the three traditional classifiers, LR, RF, and SVM. Results again showed that RF exhibited the best performance (F-score = 0.865).

**(2)** Using an auto-encoder to train the joint vector and extract the middle layer of the encoder as input to LR, RF, and SVM. The RF performed best (F-score = 0.777), which means that the auto-encoder cannot effectively extract high-level embedding information from combining features.

#### 3.3.2. Multimodal Deep Auto-Encoder

A multimodal deep auto-encoder is designed to train each feature by a specific auto-encoder, and extract the embedding as the corresponding representations. The input of each auto-encoder is the feature vectors *Seq*, *PSSM*, *SS*, and *AA*. The neurons of the corresponding auto-encoder for each feature are *Seq*: 256–128–256, *PSSM*: 256–128–256, *AA*: 512–128–512, and *SS*: 256– 128–256. After training layer by layer, we concatenate the middle layer of each auto-encoder as the input training vector of the classifiers. We tested LR, RF, and SVM on the concatenated middle layer. The performance of RF was better (F-score = 0.879) than the other two machine learning classifiers.

### 3.4. Comparison With Other Existing Tools for Predicting MPs

MoonGO (Chapple et al., 2015), MPFit (Khan and Daisuke, 2016), and DextMP (Khan et al., 2017) are existing computation methods for predicting MPs. For the purpose of comprehensive evaluation, MPFit is compared with MEL-MP. MPFit is a machine learning based model for predicting MPs. MPFit compiled six omic-based features from protein association information and tested the random combination of those six features. Because a single feature cannot cover all of the sample, MPFit used the RF to fix the missing data problem based on randomly combining the different association features. Among the single omic features tested, the highest F-score of MPFit is 0.710 obtained by gene expression (GE). When using the combined features to train the prediction model, F-scores were within a range from 0.571 to 0.784. For MPFit, the missing association annotation information is inevitable. With our proposed *de novo* prediction model, MEL-MP, the data coverage can reach 100%, which is more practical. Moreover, the F-score of MEL-MP is 0.892, which is nearly 10% higher than the omic data-based prediction of MPFit, with an F-score of 0.784. MoonGO and DextMP are entirely different types of methods compared to MEL-MP. While MoonGO is an unsupervised method based on GO annotation. MoonGO does not provide reference predictive performance. So it is not practical and meaningless to compare MEL-MP with DextMP and MoonGO.

### 3.5. Case Study

MEL-MP is conducted on 20,354 proteins. Among them, 7,250 are predicted as MPs. All the predicted MPs can be downloaded from the web server. For further explanation of the mechanisms of MPs, their distribution on chromosomes, evolution, and disease association are discussed.

#### 3.5.1. Distribution of Predicted MPs on Chromosomes

**Figure 3:**
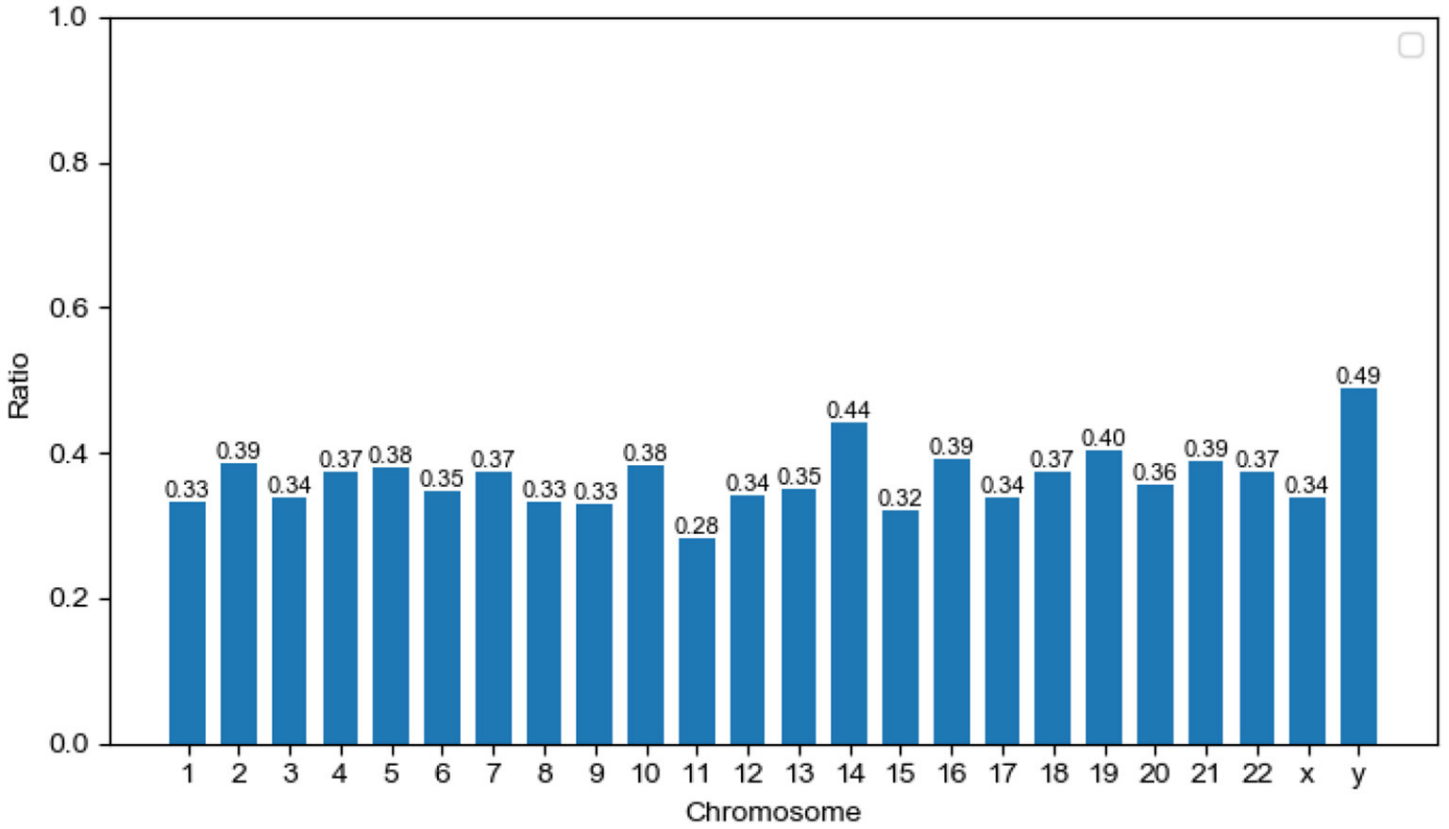
Distribution of potential moonlighting proteins (MPs) in human chromosomes. The x-axis corresponds to chromosome pairs and sex chromosomes (x and y). The y-axis measures the ratio of predicted MPs on single chromosome pairs.

The ratio of predicted MPs is shown for each human chromosome pair (Figure 3). The ratio of MPs on the Y chromosome is the highest (48.97%) compared to other chromosomes. In recent years, an increasing amount of research in this domain has focused on the Y chromosome. This chromosome not only determines gender, but is also used to study many facets of biology, including the evolution, migration, and scope for expansion of modern human (Quintana-Murci et al., 2001). We argue that the study of multitasking proteins is an important new area for Y chromosome research. The numbers of the Uniprot proteome, predicted MPs, and the ratio of predicted MPs in each chromosome are shown in Supplementary Table 4.

#### 3.5.2. Biological Evolution of Predicted MPs

We discuss the predicted MPs from the perspective of biological evolution using phylostratum (Domazet Loso and Tautz, 2010). Phylostratum is the study of classifying genes according to their ages, which represent the time since the original ancestor of the specific gene family appeared. The phylostratum is correlated with the complexity of organisms. The larger the phylostratum of a species, the more advanced the species is. Domazet Loso and Tautz (2010) generated a generate a database of phylostrata corresponding to the evolutionary relationships of the phylogenetic based on phylogenomic analyses. Cellular orgs, which is the lowest phylogenetic, have a phylostratum of 1; in opposite, the primates have a phylostratum of 19. There are 20,259 genes corresponding to these organisms. Here, we map these genes to proteins through the Uniprot database. The predicted MPs are mapped to 5,849 genes, while the predicted non-MPs (the proteins that were predicted as non-MP through MEL-MP) are mapped to 10,597 genes. We calculated the phylostratum of proteins according to the phylostratum of corresponding genes. The median and mean of the phylostratum of predicted MPs mapped genes are 1 and 2.802, respectively, whereas the median and mean of the phylostratum of predicted non-MPs are 2 and 3.822, respectively.

**Figure 4:**
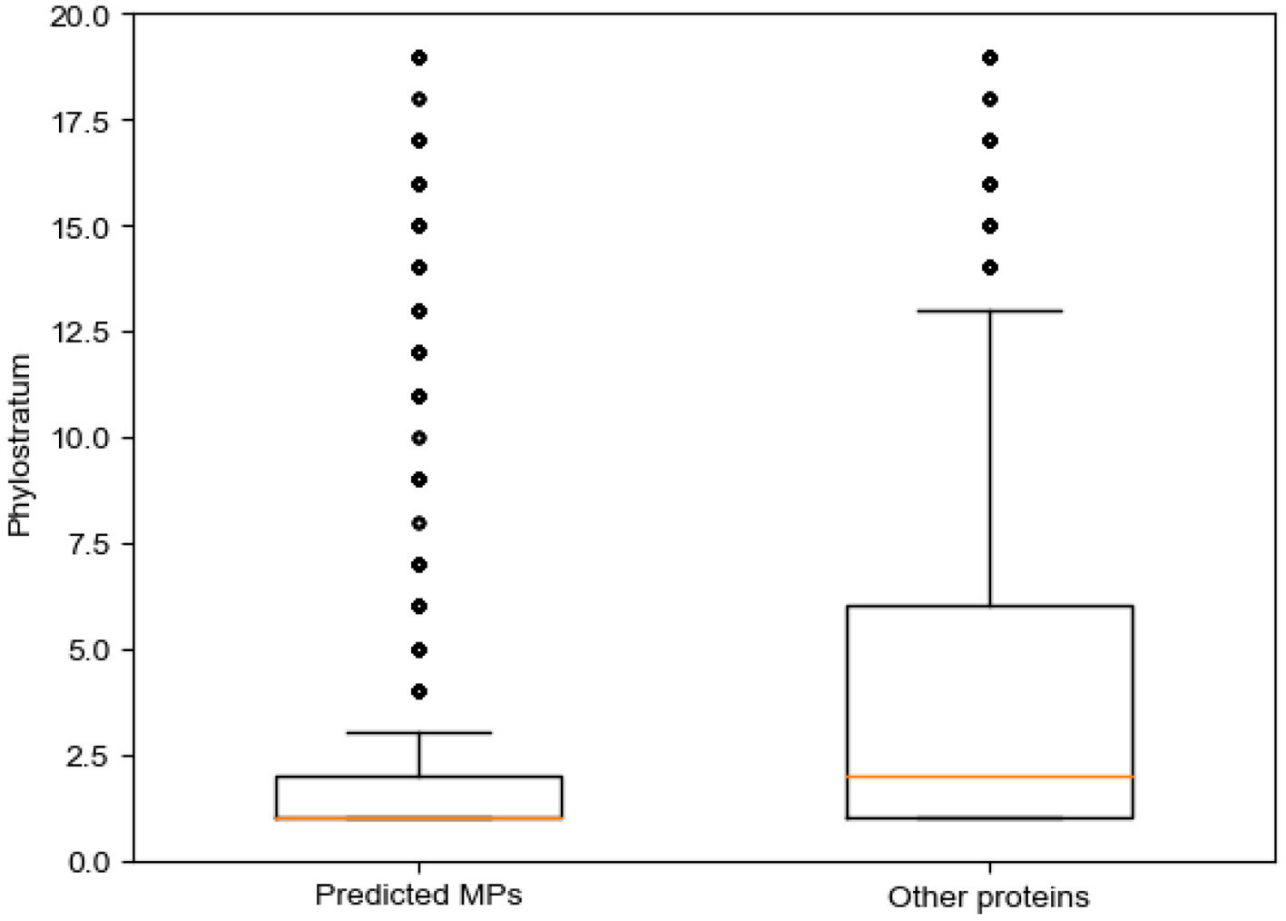
Phylostrata for predicted moonlighting proteins (MPs) and other proteins.

In addition, the hypergeometric test (HGT) is applied to analysis of the predicted MPs and non-MPs belonging to each phylostratum, respectively (shown in Supplementary Material, section 1.3). The predicted MPs is only significantly enriched in the phylostratum of 1. The archea, bacteria, etc., are the phylogenetic of cellular org. Compared with other proteins, significantly enriched in many of phylostratum except 1. Therefore, we can infer that the older the species from which a protein originates, the more functions the protein will have. A recent study found that genes with an older genetic age tend to have more functional domains (Choi et al., 2018). Therefore, our experiments further verify this view. Figure 4 shows the box plot of phylostratum of predicted MPs and predicted non-MPs.

#### 3.5.3. Diseases Association of Predicted MPs

**Figure 5:**
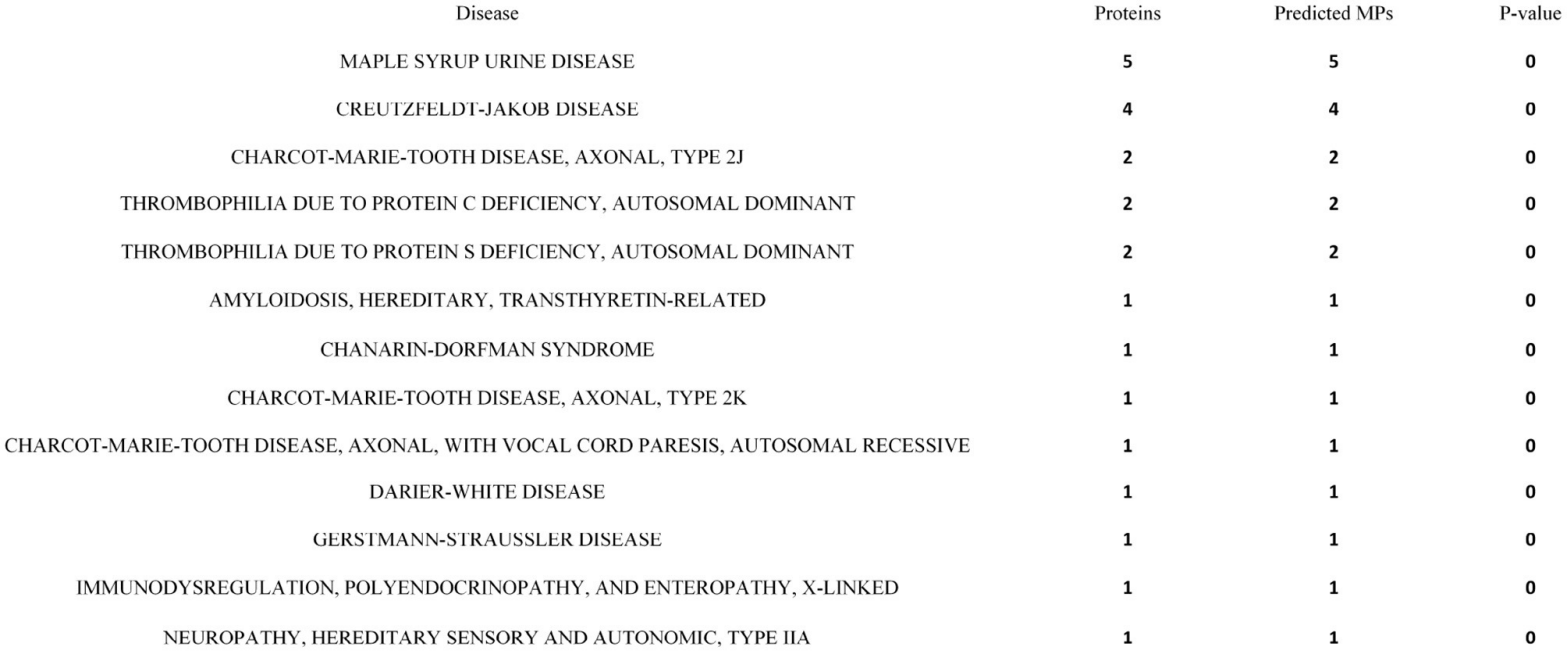
The top 13 diseases enriched by moonlighting proteins (MPs).

For the purpose of studying MP disease associations, we introduce the relationships between predicted MPs and 114 diseases in the OMIM database (Amberger et al., 2015). We downloaded the protein accession of each disease in the OMIM database from the Uniprot database. The proteins corresponding to each disease are presented in Supplementary Table 5. An HGT is applied to evaluate the relationship between predicted MPs and diseases:

(15) $$P-value=1-\sum_{i\;=\;0}^{m-1}\frac{\left(\begin{array}{c} M\\ i \end{array}\right)\left(\begin{array}{c} N-M\\ n-i \end{array}\right)}{\left(\begin{array}{c} N\\ n \end{array}\right)}$$

where *N* is the total number of Uniprot reviewed proteins, *n* is the number of proteins correlated to diseases in the OMIM database, *M* is total the number of predicted MPs, and *m* refers to the number of predicted MPs correlated to these diseases. With respect to other proteins, *M* is the number of those other proteins and *m* refers to those other proteins in diseases. There are 20,354 human proteins reviewed in the Uniprot database, 7,250 proteins are predicted as MPs, and 1,112 of the predicted MPs are mapped to diseases. The *P* − *value* is 0.00033 (*P* < 0.05), which means these diseases are significantly enriched in predicted MPs. With respect to other proteins, 1,782 are mapped to these diseases. The *P* − *value* is 0.99962 (*P* > 0.05). The significance of MPs related to disease has thus been reflected. Furthermore, the enrichment analysis process of MPs for all diseases is presented in Supplementary Material, section 1.4. Supplementary Table 7 shows the enrichment analysis of MPs for each disease. The disease with *P* < 0.05 is significant. Among 114 of OMIM diseases, MPs are enriched to 21 of OMIM diseases. The top 13 enriched diseases of MPs are shown in Figure 5, and these diseases are all correlated less than five proteins, which are all predicted as MPs. For non-MPs, 1,782 are mapped to OMIM diseases. The *P* − *value* are larger than 0.05. Therefore, the predicted MPs are significantly related to diseases.

#### 3.5.4. Functional Inference of Predicted MPs and Non-MPs

**Figure 6:**
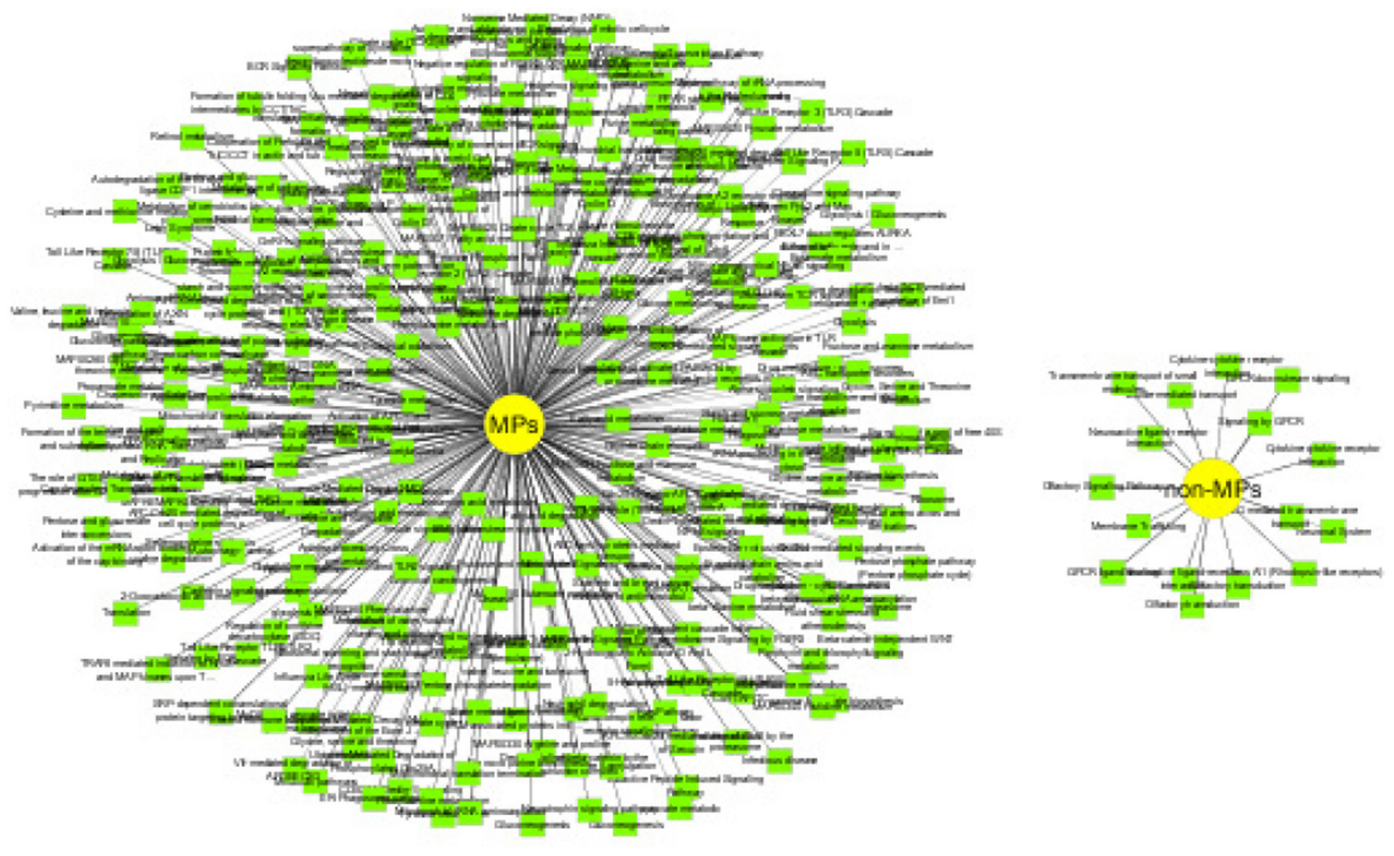
Pathway diagram of predicted moonlighting proteins (MPs) and other proteins.

**Figure 7:**
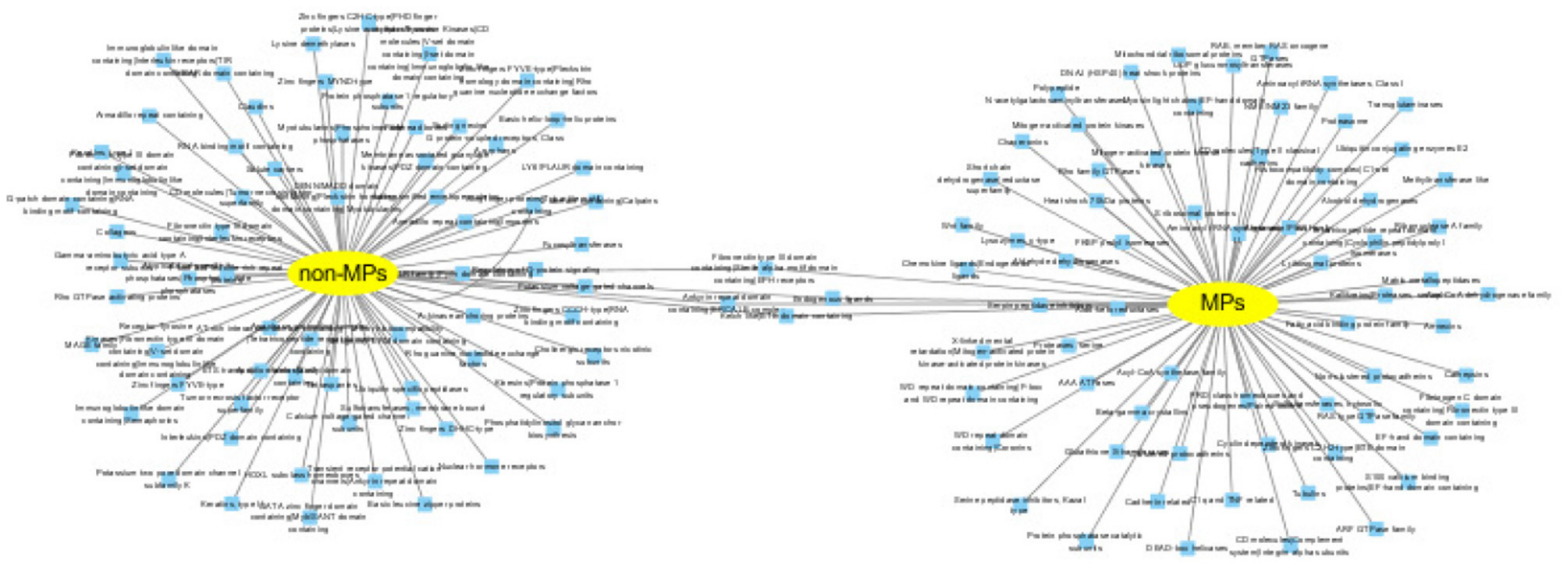
The cluster of gene family of predicted moonlighting proteins (MPs) and other proteins.

MPs have two or more different functions. We conduct functional inference on MPs and non-MPs using Toppcluster (Kaimal et al., 2010), which is an efficient and convenient enrichment analysis tool. Here, we perform the functional inference from two perspectives: biological pathways and protein families. The cutoff of *P* − *value* is set as 0.05. The detailed experiment results are shown in Supplementary Tables 8, 9. For predicted MPs, the number of the enriched pathways are 313, which is significantly higher than non-MPs with only 16 enriched pathways. The enrichment of biological pathways intuitively reflect the functional versatility of predicted MPs, which further verify the efficiency of our MEL-MP tool. The pathway diagram of MPs and non-MP is shown in Figure 6. In addition, the proteins in the same protein usually have similar function and evolutionary history. For further exploring the predicted MPs and non-MPs, the enriched protein families are analyzed. The protein family diagram of predicted MPs and non-MPs is shown in Figure 7. From Figure 7, the enriched protein families between predicted MPs and non-MPs are significantly different.

### 3.6. **Web Server**

For ease of use, we provide an MEL-MP web server, which can be accessed at: http://bmbl.sdstate.edu/mel-mp/. The source code and our results of four case studies can be downloaded from this website. The “help” interface provides the guidance for the use of MEL-MP web server. Users can input the protein name and protein sequence in fasta format. The unique job id is provided to users for downloading and recovering the prediction results.

## 4. Discussion

In this paper, we proposed a *de novo* prediction tool, MEL-MP, to identify MPs based on protein sequences. MEL-MP is the first *de novo* prediction method for MPs. It is both concise and effective. Indeed, compared with other machine learning methods, our sequence-based method is more comprehensive and universal because it can cover more MPs without missing data. It is suitable for protein-related machine learning applications and cognate studies. Further, ensemble learning has advantages in the sense that the greater the diversity of features, the better the performance of the classifiers (Kuncheva and Whitaker, 2003).

Through comparative experiments, the stacked ensemble architecture performed better than other feature fusion methods. In addition to researching proteins, the study of long non-coding RNAs (lncs) is another hot topic in bioinformatics. Moonlighting long noncoding RNAs (Mlncs) are particular lncs with two or more distinctive functions, which have significant potential in terms of application-oriented research. Currently, computing methods associated with the identification of Mlncs are based on network analysis (Lixin and Kwong-Sak, 2018). It would be prudent and useful for future research to extend the machine learning method put forward in this paper to the prediction of Mlncs.

## 5. Conclusions

In this study, we proposed a *de novo* machine learning method based on sequence for MPs prediction, named MEL-MP. We use the ensemble learning strategy to integrate the models corresponding to the four sequence features and achieve good accuracy. Moreover, our case study includes four popular perspectives, chromosome research, protein–disease correlation, biological evolution, and functional description. It also promotes the research of MPs. In addition, studying MPs will also provide some motivation for the research of Mlncs.

## Data Availability Statement

The original contributions presented in the study are included in the article/Supplementary Material, further inquiries can be directed to the corresponding author/s.

## Author Contributions

YL designed the research plan and checked and revised the manuscript. JZ collected and analyzed the data and checked and revised the manuscript. ZL and WD checked and revised the manuscript. CW, LW, and SH developed the web server. All authors contributed to the article and approved the submitted version.

## Conflict of Interest

The authors declare that the research was conducted in the absence of any commercial or financial relationships that could be construed as a potential conflict of interest. The handling editor declared a past co-authorship with the authors ZL and CW.

## Abbreviations

AA-MLP: physical and chemical properties classing by multilayer perceptron
Acf: autocorrelation coefficient function
BiLSTM: bidirectional long short-term memory neural networks
BP: biological process
CC: cellular components
GO: gene ontology
lnc: long noncoding RNAs
LR: logistic regression
MF: molecular function
Mlnc: moonlighting long noncoding RNAs
MLP: multilayer perceptron
MP: moonlighting protein
non-MP: non-moonlighting proteins
PSSM-RF: PSSM applying random forest
*rawpssm*: position-specific score matrix
RF: random forest
Seq-BiLSTM: sequence feature applying bidirectional long short-term memory neural networks
*sequence*: protein sequence
SS-ARF: auto-encoder and RF corresponding to secondary structure
SVM: support vector machine.
