# Supplementary material for: De novo Prediction of Moonlighting Proteins Using Multimodal Deep Ensemble Learning
Source: Front Genet. 2021 Mar 22;12:630379. doi: 10.3389/fgene.2021.630379 (PMC8019903; doi:10.3389/fgene.2021.630379)
Supplement: Supplementary file 1 [file Data_Sheet_1.pdf]

frontiers

Supplementary Material

1 Supplementary Data

1.1 The hyperparameter selection experiments of sub-models using deep learning

The architecture of MEL-MP contains four sub-models and a stacked ensemble model. To improve overall predicting performance, the accuracy of the four sub-models should be as high as possible. Due to the deep learning model contains many of hyperparameters, they can significantly affect accuracy. Thus, we conducted a lot of experiments to select hyperparameters for three deep learning based sub-models, Seq-BiLSTM, AA-MLP, and SS-ARF. The criteria of evaluation contain accuracy, precision, recall, and f-score. We tested all of the deep learning models using the optimizer of Adam, Adadelta, and Momentum. The details of four sub-models are shown as follows:

Seq-BiLSTM

In bidirectional long short-term memory neural networks (BiLSTM) **[1]** model, we split the Seq vector into 5, 4, 8, 10, 20 segments as the time steps, the number of inputs refers the dimension of segments, which means the number of inputs is equal to the dimension of Seq divide by time steps. When using the momentum optimizer, we set the momentum = 0.9, using the same time steps and the number of input parameter, each evaluation criterion is slightly higher than the performance of other optimizers. When the time steps are chosen to 4, then any of the criteria is the best among using other parameters. The Seq-BiLSTM achieved better accuracy. The hyperparameter selection of BiLSTM model is shown in **Table S1**:

Table S1. The performance of BiLSTM model using different hyperparameters.

Optimizer	Number of input	Time steps	Precision	Recall	F-score
Momentum	80	5	0.770	0.762	0.758
	20	20	0.763	0.759	0.759
	40	10	0.795	0.785	0.785
	50	8	0.822	0.813	0.810
	100	4	0.851	0.844	0.851
Adam	80	5	0.751	0.738	0.737
	20	20	0.743	0.734	0.732
	40	10	0.795	0.792	0.788
	50	8	0.820	0.815	0.814
	100	4	0.822	0.815	0.814
Adadelta	80	5	0.765	0.759	0.755
	20	20	0.760	0.736	0.737
	40	10	0.778	0.766	0.767
	50	8	0.805	0.794	0.795
	100	4	0.808	0.804	0.803

AA-MLP

In consideration of a neural network model of multi-Layer perceptron (MLP), we test the different layers and the different number of neurons in each layer under the above three optimizers. The highest accuracy occurs to 2 layers network, using adadelta optimizer, the number of neurons in each layer is 553, 276. The precision, recall, f-score is 0.861, 0.853, 0.853, respectively, which is not only the highest performance but also cost less time compared to 3 layers neural network. The hyperparameter selection experiments are shown in **Table S2.**

Table S2. The performance of MLP model using different hyperparameters.

Optimizer	Architecture	Precision	Recall	F-score
Adadelta	512-256	0.838	0.825	0.826
	256-128	0.818	0.816	0.814
	553-276	0.861	0.853	0.853
Adam	512-256	0.857	0.846	0.845
	256-128	0.841	0.832	0.830
	553-276	0.861	0.848	0.848
Momentum	512-256	0.850	0.839	0.838
	256-128	0.831	0.823	0.820
	553-276	0.814	0.806	0.804
Adadelta	553-276-138	0.846	0.834	0.835
	512-256-128	0.854	0.839	0.839
	256-128-64	0.827	0.820	0.820
Adam	553-276-138	0.849	0.839	0.837
	512-256-128	0.846	0.837	0.835
	256-128-64	0.828	0.820	0.818
Momentum	553-276-138	0.841	0.834	0.833
	512-256-128	0.850	0.841	0.840
	256-128-64	0.836	0.830	0.829

SS-ARF

The deep auto-encoder **[2]** contains the encoding layers and decoding layers, through the neural network is trained layer by layer, the middle layer has the high-level feature of input vectors. In the deep auto-encoder model, the number of encoding layers and decoding layers is the same. We test the architecture of 512-256-512, 256-128-256, and 512-256-128-256-128 neurons in each layer, the middle layer is the feature vector need to be classified. In this task, the deep autoencoder using the Adam optimizer, the architecture of 512-256-512 has the best ability of feature representation among others. Classified by random forest (RF), the precision, recall, F-score is 0.839, 0.837, 0.833, respectively. The hyperparameter selection experiments are shown in **Table S3**.

Table S3. The results of different hyperparameters of deep autoencoder in SS-ARF model.

Optimizer	Architecture	Classifier	Precision	Recall	F-score
Adadelta	256-128	LR	0.764	0.762	0.760
		SVM	0.790	0.790	0.786
		RF	0.790	0.790	0.786
	512-256	LR	0.749	0.745	0.744
		SVM	0.792	0.790	0.787
		RF	0.792	0.790	0.787
	512-256-128	LR	0.756	0.752	0.752
		SVM	0.793	0.790	0.785
		RF	0.793	0.790	0.785
Momentum	512-256-128	LR	0.722	0.713	0.713
		SVM	0.757	0.759	0.749
		RF	0.757	0.759	0.749
	256-128	LR	0.769	0.757	0.758
		SVM	0.791	0.790	0.782
		RF	0.791	0.790	0.782
	512-256	LR	0.678	0.664	0.664
		SVM	0.763	0.750	0.726
		RF	0.763	0.750	0.726
Adam	512-256	LR	0.749	0.738	0.738
		SVM	0.797	0.792	0.787
		RF	0.839	0.837	0.833
	256-128	LR	0.703	0.696	0.695
		SVM	0.770	0.769	0.757
		RF	0.770	0.769	0.757
	512-256-128	LR	0.739	0.731	0.732
		SVM	0.783	0.781	0.771
		RF	0.783	0.781	0.771

1.2 The research on the MPs corelation to each of chromosome.

We counted the number of proteins on each chromosome, the number of MPs and the proportion (Ratio) of MPs. Shown in **Table S4**.

Table S4. The distribution of predicted MPs in each chromosome.

Chromosome	Total numbers of proteins	Predicted moonlighting proteins	Ratio
humchr01	2067	687	0.3324
humchr02	1289	498	0.3863
humchr03	1077	364	0.3380
humchr04	765	286	0.3739
humchr05	874	332	0.3799
humchr06	1029	358	0.3479
humchr07	1004	374	0.3725
humchr08	702	233	0.3319
humchr09	810	267	0.3296
humchr10	749	286	0.3818
humchr11	1331	375	0.2817
humchr12	1033	351	0.3398
humchr13	332	116	0.3494
humchr14	727	321	0.4415
humchr15	603	194	0.3217
humchr16	841	330	0.3924
humchr17	1170	397	0.3393
humchr18	275	103	0.3745
humchr19	1434	578	0.4031
humchr20	550	196	0.3564
humchr21	254	99	0.3898
humchr22	502	188	0.3745
humchr	844	285	0.3377
humchry	47	23	**0.4894**

1.3 Biological evolution of predicted MP.

The phylostratum **[3]**, which is clustering genes according to their age. The genetic age is ranging from 1 to 19. Domazet- Loso and Tautz collected 20259 genes and their genetic age in 2010. Thence, we converted the UNIPROT **[4]** ID of the 20354 human proteins we studied into gene ID. The all UNIPROT accession are associated with 16446 genes, the MPs are associated with 5849 genes, and the non-MPs is associated with 10597 genes. Shown in **Table S5**. Furthermore, we studied the enrichment of genes corresponding to MPs and non-MPs in each genetic age using the hypergeometric test (HGT) as follow:

$$P - value = 1 - \sum_{i=0}^{m-1} \frac{\binom{M}{i}\binom{N-M}{n-i}}{\binom{N}{n}}$$

Where N = 16446, which is the number of all associated genes of all UNIPROT accession. For predicted MPs, the M is 5849, which is the number of the associated genes of predicted MPs, the n is the number of genes corresponding to UNIPROT accession at various genetic ages. And the m refers to the number of genes corresponding to the predicted MPs. For predicted non-MPs, same as predicted MPs, the M, m is the 10597 and the number of genes corresponding to the predicted non-MPs. Here, we select $P - value$ less than 0.05 to determine whether each type of protein is enriched in a certain genetic age. The results are shown in **Table S6**.

Table S5. Distribution of genes mapped to MPs and non-MPs in genetic ages ranging from 1 to 19.

	UNIPROT proteins	MPs	non-MPs
Total number	20354	7250	13104
The number of associated genes	16446	5849	10597

Table S6. The enrichment of genes corresponding to MPs and non-MPs in each genetic age.

Phylostratum (Genetic age)	Phylogenetic	Genes（MPs）P-value	Genes（nonMPs） P-value
1	Cellular org	4.19E-221	1
2	Eukaryota	1	3.74E-42
3	OpisthoKonta	0.999999998	7.68E-10
4	Holozoa (Metazoa+Allies)	0.999916369	4.79E-05
5	Metazoa	0.991251145	0.006275
6	Eumetazoa	1	8.43E-88
7	Bilateria	0.999993939	4.05E-06
8	Deuterotomia	0.995322575	0.001385
9	Chordata	0.98124205	0.011816
10	Olfactors (Craniata + Urochordata)	0.863730931	0.06533
11	Craniata (Vertebrata)	0.18506727	0.782261
12	Euteleostomi (Osteichthyes)	0.999999946	2.87E-08
13	Tetrapoda	0.62098177	0.28129
14	Amniota	0.967770952	0.019367
15	Mammalia	0.99406807	0.003528
16	Eutheria	0.708605486	0.245547
17	Boreoeutheria	0.65574872	0.251309
18	Euarchontoglires	0.254609453	0.58014
19	Primates	0.827765731	0.13163

1.4 The diseases corelation of predicted MPs.

We also applied HGT to research the predicted MPs associated with various diseases in OMIM database. OMIM collected 114 type of disease. For each type of disease, we collect the corelated proteins in UNIPROT database. In our study, 2894 of UNIPROT proteins is corresponding to the OMIM database, 1112 of predicted MPs is corresponding to OMIM database. Therefore, the N and M is 2894 and 1112 respectively. Where n is the number of proteins in each disease, m is the number of predicted MPs in each disease. Finally, we calculated the $P-value$ of MPs for each disease. If $P-value$ < 0.05, means that MPs is significantly enriched in specific disease. The results are shown in **Table S7**.

Table S7. The number of predicted MPs corresponding to the diseases in OMIM database. If the P-value less than 0.05, means that MPs are enriched in the disease.

Disease	Proteins	Predicted MPs	P-value
MAPLE SYRUP URINE DISEASE	5	5	0
CREUTZFELDT-JAKOB DISEASE	4	4	0
CHARCOT-MARIE-TOOTH DISEASE, AXONAL, TYPE 2J	2	2	0
THROMBOPHILIA DUE TO PROTEIN C DEFICIENCY, AUTOSOMAL DOMINANT	2	2	0
THROMBOPHILIA DUE TO PROTEIN S DEFICIENCY, AUTOSOMAL DOMINANT	2	2	0
AMYLOIDOSIS, HEREDITARY, TRANSTHYRETIN-RELATED	1	1	0
CHANARIN-DORFMAN SYNDROME	1	1	0
CHARCOT-MARIE-TOOTH DISEASE, AXONAL, TYPE 2K	1	1	0
CHARCOT-MARIE-TOOTH DISEASE, AXONAL, WITH VOCAL CORD PARESIS, AUTOSOMAL RECESSIVE	1	1	0
DARIER-WHITE DISEASE	1	1	0
GERSTMANN-STRAUSSLER DISEASE	1	1	0
IMMUNODYSREGULATION, POLYENDOCRINOPATHY, AND ENTEROPATHY, X-LINKED	1	1	0

NEUROPATHY, HEREDITARY SENSORY AND AUTONOMIC, TYPE IIA	1	1	0
CENTRAL CORE DISEASE OF MUSCLE	175	89	0.0002
PROTEIN Z DEFICIENCY	1163	479	0.0055
ALPHA-1-ANTITRYPSIN DEFICIENCY	5	4	0.0083
MENKES DISEASE	5	4	0.0083
BRAIN SMALL VESSEL DISEASE 1 WITH OR WITHOUT OCULAR ANOMALIES	7	5	0.0150
CHARCOT-MARIE-TOOTH DISEASE, TYPE 4A	11	7	0.0224
PELIZAEUS-MERZBACHER DISEASE	6	4	0.0340
MYOCLONIC EPILEPSY OF LAFORA	10	6	0.0437
INFLAMMATORY BOWEL DISEASE 28, AUTOSOMAL RECESSIVE	3	2	0.0566
NORRIE DISEASE	3	2	0.0566
RHEUMATOID ARTHRITIS	92	42	0.0608
PARKINSON DISEASE 6, AUTOSOMAL RECESSIVE EARLY-ONSET	17	9	0.0707
GLYCOGEN STORAGE DISEASE II	37	18	0.0740
GLYCOGEN STORAGE DISEASE III	37	18	0.0740
GLYCOGEN STORAGE DISEASE V	37	18	0.0740
PICK DISEASE OF BRAIN	15	8	0.0745
GLYCOGEN STORAGE DISEASE VI	31	15	0.0927
ALEXANDER DISEASE	45	21	0.0978

ALZHEIMER DISEASE 3	198	83	0.1309
NEURODEGENERATION WITH BRAIN IRON ACCUMULATION 1	12	6	0.1314
CHARCOT-MARIE-TOOTH DISEASE, DEMYELINATING, TYPE 1B	10	5	0.1407
CHARCOT-MARIE-TOOTH DISEASE, AXONAL, TYPE 2F	2	1	0.1476
INFLAMMATORY BOWEL DISEASE 25, AUTOSOMAL RECESSIVE	8	4	0.1499
NIEMANN-PICK DISEASE, TYPE B	8	4	0.1499
ALZHEIMER DISEASE	202	84	0.1510
ALZHEIMER DISEASE 2	200	83	0.1581
HUNTINGTON DISEASE-LIKE 1	4	2	0.1614
INFANTILE SIALIC ACID STORAGE DISEASE	4	2	0.1614
NIEMANN-PICK DISEASE, TYPE C1	4	2	0.1614
THROMBOPHILIA DUE TO PROTEIN C DEFICIENCY, AUTOSOMAL RECESSIVE	4	2	0.1614
HEMOGLOBIN H DISEASE	36	16	0.1784
CHARCOT-MARIE-TOOTH DISEASE, DOMINANT INTERMEDIATE B	16	7	0.2403
CHARCOT-MARIE-TOOTH DISEASE, DOMINANT INTERMEDIATE C	16	7	0.2403
CHARCOT-MARIE-TOOTH DISEASE, DEMYELINATING, TYPE 1C	7	3	0.2597
CHOLESTASIS, PROGRESSIVE FAMILIAL INTRAHEPATIC, 1	7	3	0.2597
FABRY DISEASE	5	2	0.2905

PIGMENTED NODULAR ADRENOCORTICAL DISEASE, PRIMARY, 1	5	2	0.2905
CHARCOT-MARIE-TOOTH DISEASE, AXONAL, TYPE 2L	3	1	0.3294
GRANULOMATOUS DISEASE, CHRONIC, AUTOSOMAL RECESSIVE, CYTOCHROME b-POSITIVE, TYPE I	3	1	0.3294
GRANULOMATOUS DISEASE, CHRONIC, AUTOSOMAL RECESSIVE, CYTOCHROME b-POSITIVE, TYPE II	3	1	0.3294
INCLUSION BODY MYOPATHY WITH EARLY-ONSET PAGET DISEASE WITH OR WITHOUT FRONTOTEMPORAL DEMENTIA 1	3	1	0.3294
MACHADO-JOSEPH DISEASE	3	1	0.3294
PEROXISOME BIOGENESIS DISORDER 1B	3	1	0.3294
TANGIER DISEASE	3	1	0.3294
ADRENOLEUKODYSTROPHY	25	10	0.3513
GAUCHER DISEASE, TYPE I	18	7	0.3822
GAUCHER DISEASE, TYPE II	18	7	0.3822
GAUCHER DISEASE, TYPE III	18	7	0.3822
CHARCOT-MARIE-TOOTH DISEASE, AXONAL, AUTOSOMAL DOMINANT, TYPE 2A2A	1	0	0.3842
CHARCOT-MARIE-TOOTH DISEASE, AXONAL, TYPE 2A1	1	0	0.3842
CHARCOT-MARIE-TOOTH DISEASE, AXONAL, TYPE 2B1	1	0	0.3842
CHARCOT-MARIE-TOOTH DISEASE, AXONAL, TYPE 2B2	1	0	0.3842
CHARCOT-MARIE-TOOTH DISEASE, TYPE 4B1	1	0	0.3842
CHARCOT-MARIE-TOOTH DISEASE, TYPE 4B2	1	0	0.3842

CHARCOT-MARIE-TOOTH DISEASE, TYPE 4H	1	0	0.3842
FARBER LIPOGRANULOMATOSIS	1	0	0.3842
GLYCOGEN STORAGE DISEASE IXa1	1	0	0.3842
GRANULOMATOUS DISEASE, CHRONIC, X-LINKED	1	0	0.3842
LEGG-CALVE-PERTHES DISEASE	1	0	0.3842
MOWAT-WILSON SYNDROME	1	0	0.3842
PARKINSON DISEASE 19A, JUVENILE-ONSET	1	0	0.3842
CEROID LIPOFUSCINOSIS, NEURONAL, 3	26	10	0.4127
MULTIPLE SCLEROSIS, SUSCEPTIBILITY TO	26	10	0.4127
INTERVERTEBRAL DISC DISEASE	6	2	0.4230
CHARCOT-MARIE-TOOTH DISEASE, DEMYELINATING, TYPE 1A	24	9	0.4472
CEROID LIPOFUSCINOSIS, NEURONAL, 1	27	10	0.4741
SURFACTANT METABOLISM DYSFUNCTION, PULMONARY, 1	9	3	0.4777
SURFACTANT METABOLISM DYSFUNCTION, PULMONARY, 2	9	3	0.4777
TAY-SACHS DISEASE	4	1	0.4975
INFLAMMATORY BOWEL DISEASE (CROHN DISEASE) 10	20	7	0.5267
INFLAMMATORY BOWEL DISEASE (CROHN DISEASE) 1	21	7	0.5944
AMYOTROPHIC LATERAL SCLEROSIS 1	96	35	0.6136
CHYLOMICRON RETENTION DISEASE	2	0	0.6209
DANON DISEASE	2	0	0.6209

GAUCHER DISEASE, ATYPICAL, DUE TO SAPOSIN C DEFICIENCY	2	0	0.6209
GLYCOGEN STORAGE DISEASE Ia	2	0	0.6209
PSEUDOHYPOPARATHYROIDISM, TYPE IA	2	0	0.6209
FRONTOTEMPORAL DEMENTIA AND_OR AMYOTROPHIC LATERAL SCLEROSIS 1	8	2	0.6511
VON WILLEBRAND DISEASE, TYPE 1	20	6	0.7026
VON WILLEBRAND DISEASE, TYPE 2	20	6	0.7026
VON WILLEBRAND DISEASE, TYPE 3	20	6	0.7026
MYOTONIC DYSTROPHY 1	29	9	0.7324
POLYCYSTIC LIVER DISEASE 2 WITH OR WITHOUT KIDNEY CYSTS	12	3	0.7401
HIRSCHSPRUNG DISEASE, SUSCEPTIBILITY TO, 1	6	1	0.7417
GLYCOGEN STORAGE DISEASE Ic	3	0	0.7667
PARKINSON DISEASE 1, AUTOSOMAL DOMINANT	19	5	0.8011
PARKINSON DISEASE 2, AUTOSOMAL RECESSIVE JUVENILE	7	1	0.8202
REFSUM DISEASE, CLASSIC	14	3	0.8501
AUTOIMMUNE DISEASE, SUSCEPTIBILITY TO, 1	56	17	0.8681
WILSON DISEASE	1451	542	0.8748
SANDHOFF DISEASE	8	1	0.8765
DEMENTIA, LEWY BODY	15	3	0.8884
FRONTOTEMPORAL DEMENTIA	34	9	0.8988

POLYCYSTIC KIDNEY DISEASE 4 WITH OR WITHOUT POLYCYSTIC LIVER DISEASE	29	7	0.9218
SPINAL MUSCULAR ATROPHY, TYPE I	51	14	0.9328
POLYCYSTIC KIDNEY DISEASE 1 WITH OR WITHOUT POLYCYSTIC LIVER DISEASE	30	7	0.9390
POLYCYSTIC KIDNEY DISEASE 2 WITH OR WITHOUT POLYCYSTIC LIVER DISEASE	30	7	0.9390
POLYCYSTIC KIDNEY DISEASE 3 WITH OR WITHOUT POLYCYSTIC LIVER DISEASE	30	7	0.9390
SPINOCEREBELLAR ATAXIA 17	55	14	0.9707
HUNTINGTON DISEASE	46	11	0.9731
PARKINSON DISEASE, LATE-ONSET	16	2	0.9756

1.5 Functional description of predicted MPs and predicted non-MPs.

Toppcluater is a powerful tool for researching the enrichment of gene list. Here, we use Toppcluster to analysis the functional description of MPs and non-MPs. The pathway and gene family are exhibited in our experiments. Shown in **Table S8** and **Table S9**.

Table S8. The pathway analysis of predicted MPs and predicted non-MPs.

MPs or non-MPs	Pathway
MPs	Cortocotropin releasing factor receptor signaling pathway
MPs	Glyoxylate metabolism and glycine degradation
MPs	Glutathione conjugation
MPs	Degradation of beta-catenin by the destruction complex
MPs	Influenza Viral RNA Transcription and Replication
MPs	Signaling by FGFR2
MPs	Glucose metabolism
MPs	Nonsense-Mediated Decay (NMD)
MPs	Antigen processing-Cross presentation
MPs	Arginine biosynthesis
MPs	Hh mutants abrogate ligand secretion
MPs	Regulation of p38-alpha and p38-beta
MPs	Formation of tubulin folding intermediates by CCT/TriC
MPs	Dectin-1 mediated noncanonical NF-kB signaling
MPs	Eukaryotic Translation Termination
MPs	AUF1 (hnRNP D0) binds and destabilizes mRNA
MPs	The citric acid (TCA) cycle and respiratory electron transport
MPs	The role of GTSE1 in G2/M progression after G2 checkpoint
MPs	Glycolysis
MPs	MAP00561 Glycerolipid metabolism
MPs	Mitochondrial translation elongation
MPs	Biosynthesis of amino acids
MPs	Glycolysis / Gluconeogenesis
MPs	valine degradation
MPs	Phase 1 - Functionalization of compounds
MPs	Pyrimidine metabolism
MPs	Histidine metabolism
MPs	Cap-dependent Translation Initiation
MPs	Metabolism of amino acids and derivatives
MPs	MAPKinase Signaling Pathway
MPs	APC/C:Cdc20 mediated degradation of Securin
MPs	MAP kinase activation in TLR cascade
MPs	Vif-mediated degradation of APOBEC3G
non-MPs	SLC-mediated transmembrane transport
MPs	Drug metabolism - cytochrome P450
MPs	CLEC7A (Dectin-1) signaling
MPs	Metabolism of carbohydrates

MPs	MAP00480 Glutathione metabolism
MPs	Galactose metabolism
MPs	Pentose Phosphate Pathway
MPs	De novo purine biosynthesis
MPs	Activation of the mRNA upon binding of the cap-binding complex and eIFs, and subsequent binding to 43S
non-MPs	Transmembrane transport of small molecules
MPs	Hedgehog signaling pathway
non-MPs	Neuronal System
MPs	Histidine metabolism
MPs	Stathmin and breast cancer resistance to antimicrotubule agents
MPs	Disease
MPs	GTP hydrolysis and joining of the 60S ribosomal subunit
MPs	Translation
MPs	Glycolysis
MPs	Toll Like Receptor 10 (TLR10) Cascade
MPs	tyrosine metabolic
non-MPs	Olfactory transduction
MPs	superpathway of conversion of glucose to acetyl CoA and entry into the TCA cycle
MPs	Pyruvate Metabolism
MPs	Regulation of mitotic cell cycle
MPs	Translation initiation complex formation
MPs	CXCR4-mediated signaling events
MPs	Long-term potentiation
MPs	Negative regulation of FGFR2 signaling
non-MPs	Cytokine-cytokine receptor interaction
MPs	Pentose phosphate pathway
MPs	rRNA processing in the nucleus and cytosol
MPs	Autodegradation of the E3 ubiquitin ligase COP1
MPs	superpathway of methionine degradation
MPs	Tyrosine metabolism
MPs	Amino acid synthesis and interconversion (transamination)
MPs	Downstream TCR signaling
non-MPs	Olfactory transduction
MPs	TCR signaling
MPs	citrate cycle
MPs	Links between Pyk2 and Map Kinases
MPs	Fructose and mannose metabolism
MPs	ErbB1 downstream signaling
MPs	Metabolism of polyamines
MPs	Neurotrophin signaling pathway
MPs	Alanine, aspartate and glutamate metabolism
MPs	Regulation of APC/C activators between G1/S and early anaphase
MPs	superpathway of purine nucleotide salvage
MPs	MAP00051 Fructose and mannose metabolism

MPs	Retinol metabolism
MPs	isoleucine degradation
MPs	Glycine, serine and threonine metabolism
MPs	Degradation of AXIN
MPs	Major pathway of rRNA processing in the nucleolus and cytosol
MPs	Arginine and proline metabolism
MPs	Regulation of ornithine decarboxylase (ODC)
MPs	Neutrophil degranulation
MPs	Valine, leucine and isoleucine degradation
MPs	Central carbon metabolism in cancer
MPs	GLI3 is processed to GLI3R by the proteasome
MPs	APC/C-mediated degradation of cell cycle proteins
MPs	MAPK signaling pathway
MPs	Glutathione metabolism
MPs	Chaperonin-mediated protein folding
MPs	Beta3 adrenergic receptor signaling pathway
MPs	Gluconeogenesis
MPs	Pentose and glucuronate interconversions
non-MPs	GPCR ligand binding
MPs	CDK-mediated phosphorylation and removal of Cdc6
MPs	Signaling by FGFR
non-MPs	Membrane Trafficking
MPs	Metabolism of xenobiotics by cytochrome P450
MPs	Cadherin signaling pathway
MPs	Citrate cycle (TCA cycle)
MPs	Hormone-sensitive lipase (HSL)-mediated triacylglycerol hydrolysis
MPs	Metabolism of xenobiotics by cytochrome P450
MPs	Ubiquitin-dependent degradation of Cyclin D1
MPs	superpathway of pyrimidine deoxyribonucleotides de novo biosynthesis
MPs	Generic Transcription Pathway
MPs	APC:Cdc20 mediated degradation of cell cycle proteins prior to satisfaction of the cell cycle checkpoint
MPs	Toll Like Receptor TLR6:TLR2 Cascade
MPs	pyrimidine metabolic
MPs	beta-Alanine metabolism
MPs	Ribosome
MPs	Vpu mediated degradation of CD4
MPs	MAP00280 Valine leucine and isoleucine degradation
MPs	pyruvate metabolic
MPs	Alanine, aspartate and glutamate metabolism
MPs	rRNA processing
MPs	Cysteine and methionine metabolism
MPs	Chemokine signaling pathway
MPs	Glycine, serine and threonine metabolism
MPs	Glycolysis / Gluconeogenesis

MPs	Fructose and mannose metabolism
MPs	MyD88 dependent cascade initiated on endosome
MPs	Fluid shear stress and atherosclerosis
MPs	tRNA Aminoacylation
MPs	Histidine, lysine, phenylalanine, tyrosine, proline and tryptophan catabolism
MPs	MAPK6/MAPK4 signaling
MPs	Porphyrin and chlorophyll metabolism
MPs	Alpha-synuclein signaling
MPs	ErbB1 downstream signaling
MPs	Activated TLR4 signalling
MPs	Steroid hormone biosynthesis
MPs	MyD88:Mal cascade initiated on plasma membrane
MPs	MAP00020 Citrate cycle TCA cycle
MPs	Genes encoding enzymes and their regulators involved in the remodeling of the extracellular matrix
MPs	APC/C:Cdh1 mediated degradation of Cdc20 and other APC/C:Cdh1 targeted proteins in late mitosis/early G1
MPs	Chemokine signaling pathway
MPs	Citrate cycle (TCA cycle)
MPs	Glucuronate pathway (uronate pathway)
MPs	MAP00640 Propanoate metabolism
MPs	Cytosolic tRNA aminoacylation
MPs	Ubiquitin-dependent degradation of Cyclin D
MPs	Toll Like Receptor 7/8 (TLR7/8) Cascade
MPs	MAP00360 Phenylalanine metabolism
MPs	Glycolysis (Embden-Meyerhof pathway), glucose => pyruvate
MPs	Leigh Syndrome
MPs	Drug metabolism - cytochrome P450
MPs	Hypoacetylaspartia
MPs	Drug metabolism - other enzymes
MPs	Gluconeogenesis, oxaloacetate => fructose-6P
MPs	Nonsense Mediated Decay (NMD) independent of the Exon Junction Complex (EJC)
MPs	Phase II conjugation
MPs	p53-Independent G1/S DNA damage checkpoint
MPs	Influenza Infection
MPs	MAP00251 Glutamate metabolism
MPs	Tyrosine metabolism
MPs	Glycolysis, core module involving three-carbon compounds
MPs	Angiotensin II mediated activation of JNK Pathway via Pyk2 dependent signaling
MPs	butanoate metabolic
MPs	Phagosome
MPs	Trk receptor signaling mediated by the MAPK pathway
non-MPs	Signaling by GPCR
MPs	Metabolic pathways

MPs	Butanoate metabolism
non-MPs	Cytokine-cytokine receptor interaction
MPs	Nitric Oxide Signaling Pathway
MPs	ABC transporter disorders
MPs	Formation of the ternary complex, and subsequently, the 43S complex
MPs	Epstein-Barr virus infection
MPs	Selenoamino acid metabolism
MPs	Pentose and glucuronate interconversions
MPs	Phenylalanine metabolism
MPs	Regulation of Apoptosis
MPs	Cysteine and methionine metabolism
MPs	Porphyrin and chlorophyll metabolism
MPs	Proteasome
MPs	Gluconeogenesis
MPs	Amino sugar and nucleotide sugar metabolism
MPs	Bioactive Peptide Induced Signaling Pathway
MPs	Fatty acid degradation
MPs	Butanoate metabolism
MPs	arginine and proline metabolic
MPs	C-type lectin receptors (CLRs)
MPs	2-Hydroxyglutric Aciduria (D And L Form)
MPs	pentose phosphate
MPs	Purine metabolism
MPs	Mitochondrial tRNA aminoacylation
MPs	p53-Independent DNA Damage Response
MPs	Carbon metabolism
MPs	Fatty acid metabolism
MPs	Drug metabolism - other enzymes
MPs	ER-Phagosome pathway
MPs	valine, leucine and isoleucine degradation
MPs	Ascorbate and aldarate metabolism
MPs	Toll Like Receptor 2 (TLR2) Cascade
MPs	TRIF-mediated TLR3/TLR4 signaling
non-MPs	Neuroactive ligand-receptor interaction
MPs	Eukaryotic Translation Elongation
MPs	Guanine ribonucleotide biosynthesis IMP => GDP,GTP
MPs	Alpha-synuclein signaling
MPs	Biological oxidations
MPs	ABC-family proteins mediated transport
MPs	Glutathione metabolism
MPs	MAP00410 beta Alanine metabolism
MPs	glycolysis pathway
MPs	Steroid hormone biosynthesis
MPs	Phenylalanine metabolism
MPs	Thromboxane A2 receptor signaling

MPs	Galactose metabolism
MPs	Starch and sucrose metabolism
MPs	Amino sugar and nucleotide sugar metabolism
MPs	SRP-dependent cotranslational protein targeting to membrane
MPs	MAP00970 Aminoacyl tRNA biosynthesis
MPs	Pentose phosphate pathway
MPs	MAP00340 Histidine metabolism
MPs	PPAR signaling pathway
MPs	SCF-beta-TrCP mediated degradation of Emi1
MPs	glycolysis/gluconeogenesis
MPs	Retinol metabolism
MPs	Signaling by FGFR3
MPs	Autophagy - animal
MPs	Autodegradation of Cdh1 by Cdh1:APC/C
MPs	Mitochondrial translation
MPs	Aminoacyl-tRNA biosynthesis
MPs	Glucuronidation
MPs	MAP00650 Butanoate metabolism
non-MPs	GPCR downstream signaling
MPs	Pyruvate metabolism
MPs	Ensemble of genes encoding ECM-associated proteins including ECM-affiliated proteins, ECM regulators and secreted factors
MPs	Purine ribonucleoside monophosphate biosynthesis
MPs	Insulin signaling pathway
MPs	5-Hydroxytryptamine degradation
MPs	Eukaryotic Translation Initiation
MPs	Cooperation of Prefoldin and TriC/CCT in actin and tubulin folding
MPs	beta-Alanine metabolism
MPs	leucine degradation
MPs	Neurotrophin signaling pathway
MPs	glycolysis
MPs	Ethanol oxidation
MPs	fructose and mannose metabolic
MPs	Arginine and proline metabolism
MPs	MAP00620 Pyruvate metabolism
MPs	Beta-catenin independent WNT signaling
MPs	Protein folding
MPs	APC/C:Cdc20 mediated degradation of mitotic proteins
MPs	MAP00260 Glycine serine and threonine metabolism
non-MPs	Neuroactive ligand-receptor interaction
MPs	Mitochondrial translation initiation
MPs	Tetrahydrobiopterin (BH4) synthesis, recycling, salvage and regulation
MPs	gamma-linolenate biosynthesis
MPs	Long-term potentiation
MPs	Genes related to regulation of the actin cytoskeleton
MPs	Arachidonic acid metabolism

MPs	BCR Signaling Pathway
MPs	Leucine degradation, leucine => acetoacetate + acetyl-CoA
MPs	MAP00710 Carbon fixation
MPs	Metabolism of water-soluble vitamins and cofactors
MPs	Propanoate metabolism
MPs	Toll Like Receptor 5 (TLR5) Cascade
MPs	CXCR4-mediated signaling events
MPs	Innate Immune System
MPs	T Cell Receptor Signaling Pathway
MPs	Purine metabolism
MPs	Pentose phosphate pathway (Pentose phosphate cycle)
MPs	Propanoate metabolism
MPs	Pyrimidine metabolism
MPs	Activation of APC/C and APC/C:Cdc20 mediated degradation of mitotic proteins
MPs	Ribosome
MPs	Regulation of activated PAK-2p34 by proteasome mediated degradation
MPs	Pyruvate metabolism
MPs	Toll Like Receptor TLR1:TLR2 Cascade
MPs	Insulin signaling pathway
MPs	Influenza Life Cycle
MPs	fatty acid beta-oxidation (peroxisome)
MPs	Negative regulation of FGFR3 signaling
MPs	FBXL7 down-regulates AURKA during mitotic entry and in early mitosis
MPs	Regulation of mRNA stability by proteins that bind AU-rich elements
MPs	L13a-mediated translational silencing of Ceruloplasmin expression
MPs	Chemical carcinogenesis
MPs	Degradation of GLI1 by the proteasome
MPs	Viral mRNA Translation
MPs	Activation of Matrix Metalloproteinases
MPs	NIK-->noncanonical NF-kB signaling
MPs	Glycine, Serine and Threonine Metabolism
MPs	Branched-chain amino acid catabolism
MPs	MyD88 cascade initiated on plasma membrane
MPs	MAP00010 Glycolysis Gluconeogenesis
MPs	Purine metabolism
MPs	Degradation of GLI2 by the proteasome
MPs	MAP00350 Tyrosine metabolism
MPs	MAP00380 Tryptophan metabolism
MPs	Valine, leucine and isoleucine degradation
MPs	Valine, Leucine and Isoleucine Degradation
MPs	Parkinson disease
non-MPs	Vesicle-mediated transport
MPs	Metabolism of nucleotides
MPs	Cdc20:Phospho-APC/C mediated degradation of Cyclin A

MPs	Nonsense Mediated Decay (NMD) enhanced by the Exon Junction Complex (EJC)
MPs	Toll Like Receptor 3 (TLR3) Cascade
MPs	starch and sucrose metabolic
MPs	Ubiquitin Mediated Degradation of Phosphorylated Cdc25A
MPs	Ras Pathway
MPs	MAP00252 Alanine and aspartate metabolism
MPs	Selenocysteine synthesis
non-MPs	Class A/1 (Rhodopsin-like receptors)
MPs	MAP00030 Pentose phosphate
MPs	Ascorbate and aldarate metabolism
MPs	Toll Like Receptor 9 (TLR9) Cascade
non-MPs	Olfactory Signaling Pathway
MPs	Peptide chain elongation
MPs	Formation of a pool of free 40S subunits
MPs	Glyoxylate and dicarboxylate metabolism
MPs	Mitochondrial translation termination
MPs	Ribosomal scanning and start codon recognition
MPs	MAPK signaling pathway
MPs	2-Oxocarboxylic acid metabolism
MPs	MAP00071 Fatty acid metabolism
MPs	Thromboxane A2 receptor signaling
MPs	Infectious disease
MPs	PPAR signaling pathway
MPs	MAP00330 Arginine and proline metabolism
MPs	Wnt signaling pathway
MPs	MyD88-independent TLR3/TLR4 cascade
MPs	GnRH signaling pathway
MPs	TRAF6 mediated induction of NFkB and MAP kinases upon TLR7/8 or 9 activation

Table S9. The gene family cluster of predicted MPs and predicted non-MPs.

MPs or non-MPs	Gene family cluster
MPs	Ring finger proteins|WD repeat domain containing
MPs	Aminoacyl tRNA synthetases, Class I
MPs	Argonaute/PIWI family
MPs	Fibrinogen C domain containing|Fibronectin type III domain containing
MPs	Ankyrin repeat domain containing|BRCA1 B complex
MPs	Annexins
MPs	Proteasome
MPs	Ubiquitin conjugating enzymes E2
MPs	Sulfotransferases, cytosolic
MPs	UDP glucuronosyltransferases
MPs	Polypeptide N-acetylgalactosaminyltransferases

MPs	Mitogen-activated protein kinase kinases
MPs	DEAD-box helicases
MPs	Protein phosphatase catalytic subunits
MPs	Cadherin related
MPs	DNAJ (HSP40) heat shock proteins
MPs	Cyclin dependent kinases
MPs	Histocompatibility complex|C1-set domain containing
MPs	ARF GTPase family
MPs	CD molecules|Complement system|Integrin alpha subunits
MPs	Tubulins
MPs	S ribosomal proteins
MPs	Aminoacyl tRNA synthetases, Class II
MPs	Matrix metallopeptidases
MPs	WD repeat domain containing|F-box and WD repeat domain containing
MPs	WD repeat domain containing|Coronins
MPs	Kelch like|BTB domain containing
MPs	Heat shock 70kDa proteins
MPs	Transglutaminases
MPs	CD molecules|Type II classical cadherins
MPs	Methyltransferase like
MPs	Non-clustered protocadherins
MPs	Proteases, serine
MPs	X-linked mental retardation|Mitogen-activated protein kinase-activated protein kinases
MPs	S100 calcium binding proteins|EF-hand domain containing
MPs	Aldehyde dehydrogenases
MPs	Rho family GTPases
MPs	Glutathione S-transferases
MPs	Clustered protocadherins
MPs	Mitochondrial ribosomal proteins
MPs	Short chain dehydrogenase/reductase superfamily
MPs	RAS type GTPase family
MPs	Aldo-keto reductases
MPs	Wnt family
MPs	AAA ATPases
MPs	Zinc fingers C2H2-type|BTB domain containing
MPs	Endogenous ligands
MPs	Mitogen-activated protein kinases
MPs	Ribonuclease A family
MPs	Kallikreins|Proteases, serine
MPs	EF-hand domain containing
MPs	RAB, member RAS oncogene GTPases
MPs	C1q and TNF related
MPs	Serine peptidase inhibitors, Kazal type
MPs	Myosin light chains|EF-hand domain containing

MPs	FKBP prolyl isomerases
MPs	Acyl-CoA dehydrogenase family
MPs	Chaperonins
MPs	Fatty acid binding protein family
MPs	NME/NM23 family
MPs	Serpin peptidase inhibitors
MPs	Acyl-CoA synthetase family
MPs	Cathepsins
MPs	Beta-gamma crystallins
MPs	Lysozymes, c-type
MPs	Tetratricopeptide repeat domain containing|Cyclophilin peptidylprolyl isomerases
MPs	Alcohol dehydrogenases
MPs	Chemokine ligands|Endogenous ligands
MPs	L ribosomal proteins
MPs	PRD class homeoboxes and pseudogenes|Paired boxes
MPs	Fibronectin type III domain containing|Sterile alpha motif domain containing|EPH receptors
non-MPs	HOXL subclass homeoboxes
non-MPs	Ring finger proteins|Tripartite motif containing
non-MPs	Zinc fingers FYVE-type|Pleckstrin homology domain containing|Rho guanine nucleotide exchange factors
non-MPs	Sorting nexins
non-MPs	Myotubularins|Phosphoinositide phosphatases
non-MPs	Keratins, type I
non-MPs	Solute carriers
non-MPs	MAGE family
non-MPs	Gamma-aminobutyric acid type A receptor subunits
non-MPs	Fibronectin type III domain containing|Interleukin receptors
non-MPs	Ubiquitin specific peptidases
non-MPs	RNA binding motif containing
non-MPs	Collagens
non-MPs	Fibronectin type III domain containing|I-set domain containing|Immunoglobulin like domain containing
non-MPs	Immunoglobulin like domain containing|Semaphorins
non-MPs	Transient receptor potential cation channels|Ankyrin repeat domain containing
non-MPs	Claudins
non-MPs	CD molecules|Tumor necrosis factor superfamily
non-MPs	G-patch domain containing|RNA binding motif containing
non-MPs	Nuclear hormone receptors
non-MPs	Tumor necrosis factor receptor superfamily
non-MPs	NLR family|Pyrin domain containing
non-MPs	Armadillo repeat containing|Importins
non-MPs	A-kinase anchoring proteins
non-MPs	Interleukins|PDZ domain containing

non-MPs	Phosphatidylinositol glycan anchor biosynthesis
non-MPs	ETS transcription factor family
non-MPs	Basic helix-loop-helix proteins
non-MPs	AT-rich interaction domain containing
non-MPs	Atypical dual specificity phosphatases|Phosphoinositide phosphatases
non-MPs	Regulators of G-protein signaling
non-MPs	Fucosyltransferases
non-MPs	Armadillo repeat containing
non-MPs	G protein-coupled receptors, Class A orphans
non-MPs	Cholinergic receptors nicotinic subunits
non-MPs	Rho GTPase activating proteins
non-MPs	LY6/PLAUR domain containing
non-MPs	Calcium voltage-gated channel subunits
non-MPs	Fibronectin type III domain containing|Sterile alpha motif domain containing|EPH receptors
non-MPs	Tetraspanins
non-MPs	Forkhead boxes
non-MPs	Potassium two pore domain channel subfamily K
non-MPs	Receptor Tyrosine Kinases|Fibronectin type III domain containing|V-set domain containing|Immunoglobulin like domain containing
non-MPs	Zinc fingers FYVE-type
non-MPs	C2 domain containing|Calpains
non-MPs	Endogenous ligands
non-MPs	F-BAR domain containing
non-MPs	Zinc fingers C2HC-type|PHD finger proteins|Lysine acetyltransferases
non-MPs	Kinesins|Protein phosphatase 1 regulatory subunits
non-MPs	Ankyrin repeat domain containing|BRCA1 B complex
non-MPs	Lysine demethylases
non-MPs	Immunoglobulin like domain containing|Interleukin receptors|TIR domain containing
non-MPs	Anaphase promoting complex |Tetratricopeptide repeat domain containing
non-MPs	Kelch like|BTB domain containing
non-MPs	Late cornified envelope proteins
non-MPs	Minor histocompatibility antigens|FERM domain containing
non-MPs	Ring finger proteins|Tripartite motif containing
non-MPs	GATA zinc finger domain containing|Myb/SANT domain containing
non-MPs	Membrane associated guanylate kinases|PDZ domain containing
non-MPs	DENN/MADD domain containing|Pleckstrin homology domain containing|Myotubularins
non-MPs	Zinc fingers MYND-type
non-MPs	Potassium voltage-gated channels
non-MPs	Basic leucine zipper proteins
non-MPs	Rho guanine nucleotide exchange factors
non-MPs	Zinc fingers CCCH-type|RNA binding motif containing
non-MPs	Sulfotransferases, membrane bound
non-MPs	Zinc fingers DHHC-type

non-MPs	F-box and leucine rich repeat proteins
non-MPs	Receptor Tyrosine Kinases|CD molecules|V-set domain containing|I-set domain containing|Immunoglobulin like domain containing
non-MPs	Apolipoproteins|Sushi domain containing
non-MPs	Protein phosphatase 1 regulatory subunits
non-MPs	Keratins, type II

References

[1]. Hochreiter, S. and Schmidhuber, J. (1997). Long short-term memory. *Neural Computation* 9, 1735–1780.

[2]. Vincent, P., Larochelle, H., Lajoie, I., Bengio, Y., and Manzagol, P. A. (2010). Stacked denoising autoencoders: Learning useful representations in a deep network with a local denoising gcriterion. *Journal of Machine Learning Research* 11, 3371–3408.

[3]. Joanna S Amberger, Carol A Bocchini, François Schiettecatte, Alan F Scott, Ada Hamosh. (2014). OMIM.org: Online Mendelian Inheritance in Man (OMIM®), an online catalog of human genes and genetic disorders. *Nucleic Acids Res* 43. D789-98.

[4]. Kim, S., Thiessen, E. E., Bolton, and Chen, J. (2014). Activities at the universal protein resource (uniprot). *Nucleic Acids Research.*

[5]. Vivek Kaimal, Eric E Bardes, Scott C Tabar, Anil G Jegga, Bruce J Aronow. (2010). ToppCluster: a multiple gene list feature analyzer for comparative enrichment clustering and network-based dissection of biological systems. *Nucleic Acids Res* 38. W96-102.
